# Sources of mismeasurement of RNA knockdown by DNAzymes and XNAzymes

**DOI:** 10.1039/d5cb00182j

**Published:** 2025-09-08

**Authors:** Maria J. Donde, Alicia Montulet, Alexander I. Taylor

**Affiliations:** a Department of Chemistry, King′s College London Britannia House, 7 Trinity Street London SE1 1DB UK alex.taylor@kcl.ac.uk; b Cambridge Institute for Therapeutic Immunology and Infectious Disease (CITIID), University of Cambridge, Cambridge Biomedical Campus Cambridge CB2 0AW UK

## Abstract

RNA-cleaving oligonucleotide catalysts composed of DNA and/or nucleic acid analogues (DNAzymes, modified DNAzymes and XNAzymes) are promising agents for specific knockdown of disease-associated RNAs. However, we and others have identified discrepancies between their apparent activity *in vitro versus* when transfected into cells. Here, using examples of catalysts targeting the codon 12 region of *KRAS* RNA – an unmodified DNAzyme based on the classic “10–23” motif, a modified DNAzyme (“10–23_v46”) or an XNAzyme (“FR6_1_KRas12B”) – we examine confounding effects including unintended activity during standard RNA work-up steps, leading to mismeasurement of knockdown. We find that catalysts are not irreversibly denatured by typical cell lysis reagents, nor fully degraded by typical DNase treatments, exacerbated by nuclease resistant modification chemistries. In standard RT-qPCR workflows, DNAzymes and XNAzymes were found to be capable of cleaving their target RNAs during (1) DNase treatment and (2) reverse transcription (RT) reactions, in both instances with enhanced rates compared with under quasi-physiological conditions, producing cleavage-dependent false positives. Furthermore, catalysts were found to site-specifically inhibit cDNA synthesis (*i.e.* producing cleavage-independent false positives) and in the case of DNAzymes also had the capacity to act as primers during RT, leading to an enhancement of target site cDNA as judged by digital PCR, producing (cleavage-independent) false negatives. These effects could be broadly mitigated by purification to remove catalysts at the point of RNA extraction, under denaturing conditions. We recommend that studies of oligo catalysts in cells must include a 0 h timepoint after catalyst delivery or transfection to assess the collective impact of these mismeasurements on a case by case basis.

## Abstract 2

Nucleic acid catalysts can carry over into cellular RNA preparations following transfection, causing false positive and false negative knockdown measurements.

## Graphical abstract

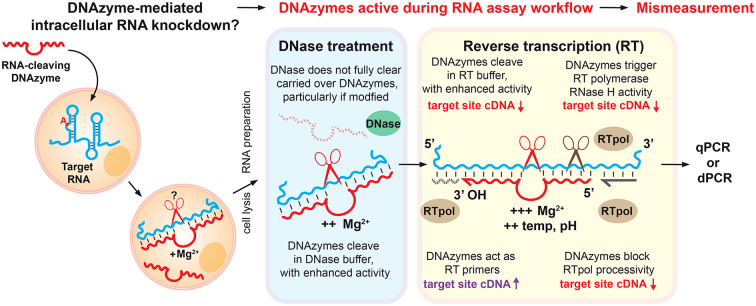

## Introduction

Enzymatic RNA cleavage mediated by nucleic acid catalysts is site-specific and remarkably selective. Among the variety of catalytic motifs identified in nature or in laboratory directed evolution experiments, those with a capacity for modular ‘reprogramming’ of Watson–Crick complementarity with a target RNA are of particular interest for rational design of specific, bespoke catalytic agents for precise modulation of disease-associated RNAs.^1–13^ Several groups are exploring allele-specific knockdown of cancer-associated mutant mRNAs encoding ‘undruggable’ oncogenes like *KRAS*^14–18^ as a proof-of-concept, although in principle such platforms could be used to modulate virtually any RNA of interest without the need for exogenous peptide-based components or co-option of host machinery, offering substantially reduced off-target effects compared with other RNA technologies.

However, RNA-cleaving catalysts composed of unmodified RNA (ribozymes) or DNA (DNAzymes) are susceptible to serum and intracellular nucleases and have limited capacity to invade heavily structured RNA targets. Classic DNAzymes like “10–23” and “8–17” may be unable to fully adopt their catalytically active states when engaging long all-RNA targets under physiologically relevant conditions (*e.g.* <1 mM free Mg^2+^)^19–22^ and may be inhibited by intracellular components including other nucleic acids or nucleotides.^20^

To address these challenges, one approach is to chemically modify pre-selected DNAzymes, which consist of ‘binding arm’ or guide strand sequences (complementary to target RNA) and a catalytic core motif, to identify which positions can tolerate modification or replacement with nucleic acid analogues with advantageous properties.^13,23–25^ Although extensive replacement of core residues is challenging without impacting activity, recent modified DNAzymes have been described using natural ribofuranose or backbone modifications, such as phosphorothioate (PS) linkages, 2′-*O*-methyl-RNA (2′OMe-RNA)^26,27^ and/or a variety of non-natural chemistries aka xeno-nucleic acids (XNAs): 2′-methoxyethyl (2′MOE-RNA),^18,28^ locked nucleic acid (LNA)^29–34^ and threose nucleic acid (TNA).^16,35^ Modified DNAzymes show encouraging improvements in catalytic activity in low [Mg^2+^], although long, structured RNAs still appear to be challenging compared to short substrates typically used for characterisation,^36^ and the presence of physiologically-relevant levels of ATP may reduce their activity at least two-fold.^18^ Alternatively, we and others have described the elaboration of fully-modified catalytic motifs (XNAzymes), selected *de novo* from XNA sequence libraries, with no DNA or RNA positions remaining and thus improved properties ‘built in’.^37–43^ “FR6_1”, a recent modular XNAzyme composed of 2′-deoxy-2′-fluoroarabinose nucleic acid (FANA),^17^ an XNA chemistry capable of stabilising secondary structures, was used to engineer a series of catalysts targeting disease-associated mRNA,^17^ non-coding RNAs^44^ and viral genomic RNA^45^ – and out-performs DNAzyme equivalents under physiological conditions.^17,44^

In addition to engineering catalysts with improved activity *in vitro*, crucial next steps in the development of clinical applications of this technology must be to establish frameworks for the rigorous assessment of their activity and fate inside cells. Although several studies including clinical trials have reported cellular or *in vivo* observations following introduction of catalysts, comparisons between active DNAzymes and catalytically dead control molecules reveal similar results.^46–50^ Collectively, these results suggest that recruitment of host cell silencing machinery (*i.e.* antisense effects), steric blocking of translation or other cytotoxic effects may be the major mechanism(s) responsible for intracellular activity. This modality is less specific than host-independent catalytic RNA cleavage as intended – for example, we have shown that allele-selectivity of a 10–23-derived DNAzyme *in vitro* does not persist when transfected into cells^17^ – so future development of catalysts with solely intrinsic activity would be beneficial for minimising off-target effects. When studying such effects with modified DNAzymes in particular, we have previously highlighted the need for rigorous design and use of control molecules, as modification chemistries and pairing of residues including, counterintuitively, those comprising a DNAzyme′s catalytic core, can potentially enhance antisense effects.^36^

**Fig. 1:**
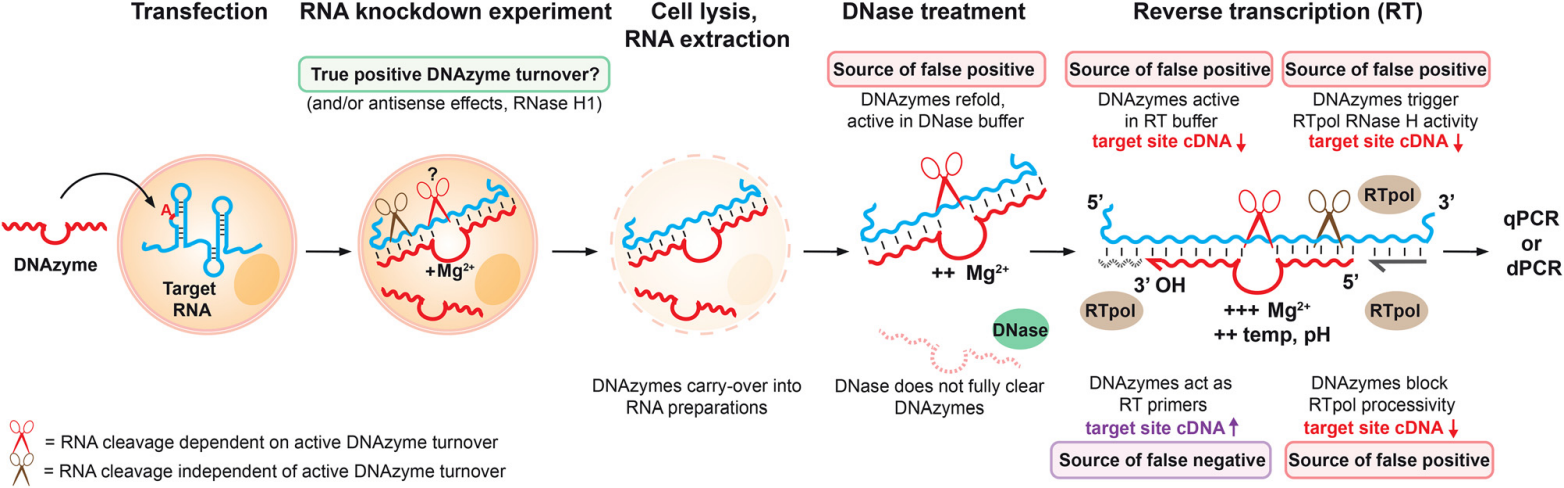
**Overview of steps in a typical assay workflow to measure DNAzyme-mediated RNA knockdown showing potential sources of misinterpretation or mismeasurement**. Diagram showing transfection or uptake of allele-specific RNA-cleaving DNAzymes intended to induce knockdown of a mutant RNA target, followed by subsequent measurement of RNA levels. Intrinsic catalytic activity of the DNAzyme (typically Mg^2+^-dependent) is represented by red scissors. Less-specific effects that directly or indirectly reduce RNA (or measured cDNA), such as antisense mechanisms, are represented by brown scissors. DNAzymes (particularly if modified with nuclease-resistant analogues) could persist through RNA assay workflows, leading to catalytic activity during these steps, potentially enhanced by favourable conditions used during workflows, as well as unintended interactions with reverse transcriptases.

Further to the question of *in vivo* mechanisms of nucleic acid catalysts, we and others^17,36,51^ have also considered the possibility that transfected DNAzymes or XNAzymes could carry over into lysates and/or RNA preparations and catalyse RNA cleavage *ex vivo* or otherwise affect the measurement of target RNA levels, a problem previously observed in studies of RNA-cleaving ribozymes.^52–54^ If this occurs, standard methods to quantify target RNA such as reverse transcription and quantitative PCR (RT-qPCR) would misleadingly indicate catalyst-dependent knockdown (and no knockdown with inactive controls, as expected) even when catalysts had failed to cleave RNA inside the cell. However, to our knowledge no detailed examination of the influence of modification chemistries on these potential pitfalls has been made. Here, using recent reported examples of unmodified and modified DNAzymes and a FANA XNAzyme, we characterise sources of false positive and false negative RNA cleavage activity during each stage of a typical RNA knockdown RT-qPCR assay workflow (Fig. 1).

## Results

### Catalysts capable of cleaving RNA transcripts under quasi-physiological conditions

**Fig. 2:**
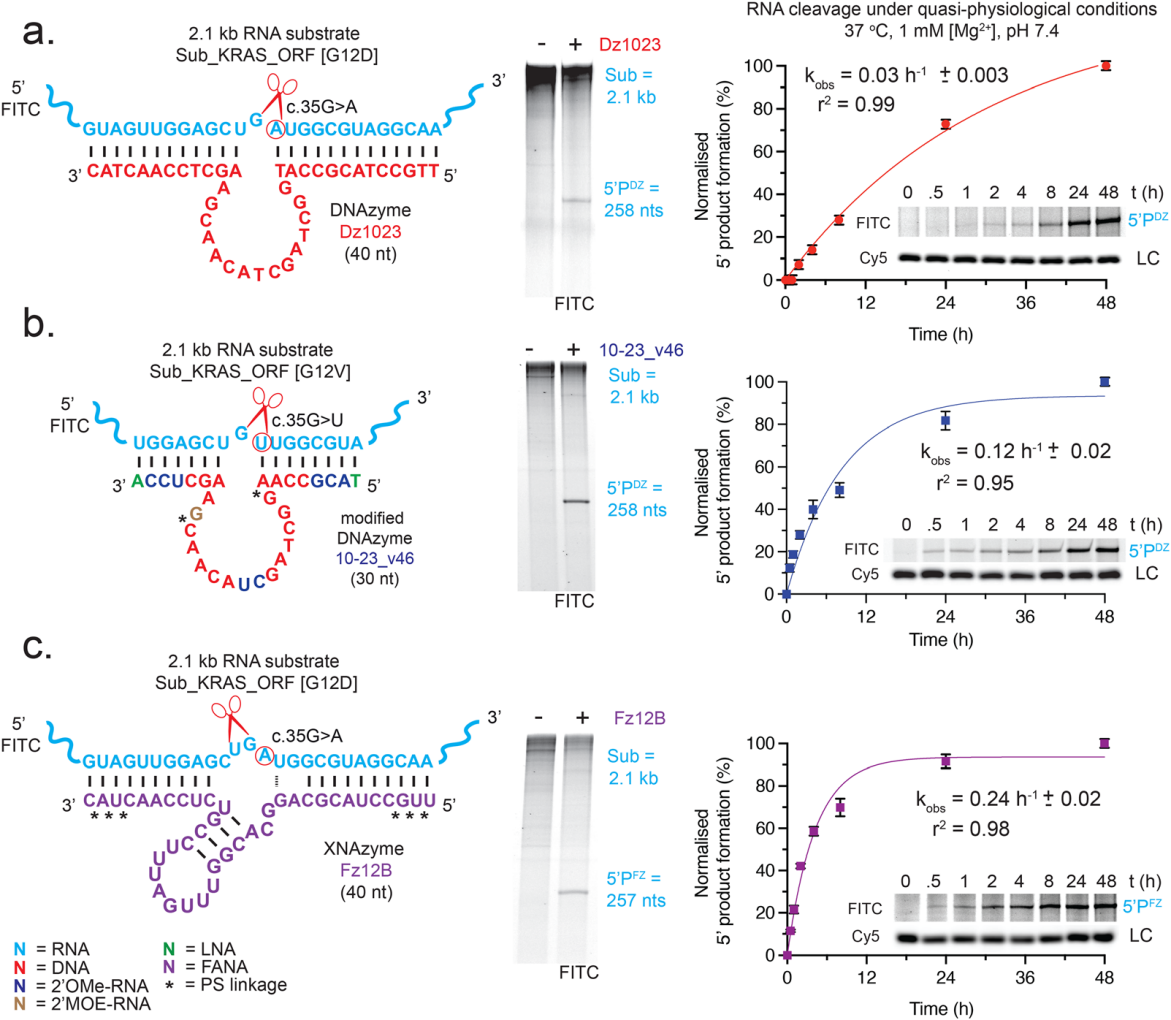
**DNAzyme and XNAzyme oligonucleotide catalysts cleave long *KRAS* transcripts slowly under physiological conditions**. RNA-cleaving catalysts and their RNA substrates, human *KRAS* RNA transcripts, used in this study; (a) unmodified DNAzyme Dz1023, (b) modified DNAzyme 10-23_v46, and (c) XNAzyme Fz12B. Red scissors indicate site of cleavage and red circles indicates RNA base (equivalent to *KRAS* c.35) that determines selectivity of cleavage between wild-type (c.35G) and mutant sequences as shown. Urea-PAGE gels (centre) show examples of single-turnover cleavage of FITC-labelled *KRAS* transcripts (1 μM) by each catalyst (5 μM) under quasi-physiological conditions (37 °C, 1 mM Mg^2+^, pH 7.4; 15 h) and graphs and example Urea-PAGE gels (right) show timecourses of the same reactions. Data and error bars represent mean ± SEM of 5′ product (5′P) formation normalised to a Cy5-labelled DNA oligo loading control (LC) in three independent experiments, with fit used to calculate apparent rate constant (*k*_obs_).

First, we chose the following three RNA-cleaving oligo catalysts as model systems, which have overlapping target sites in the mRNA sequence encoding the oncogene *KRAS*, and verified their activity under quasi-physiological conditions using their reported short (20 or 30 nt) RNA substrates (Fig. S1) as well as using more ‘realistic’ 2.1 kb synthetic transcripts, which contain the complete open reading frame (ORF) of the *KRAS* mRNA (Fig. 2); for clarity, we use HGVS coding sequence nomenclature to define residues across different RNA substrates. We reasoned that allele-specific catalysts – *i.e.* those capable of hybridising with wild-type and mutant substrates but only cleaving one of them – would allow us to disentangle potential mismeasurement effects that depend on RNA binding but which may or may not be dependent on catalyst-mediated cleavage. This is an important distinction as typical controls, non-binding or binding but catalytically inactive molecules, would fail to account for false positive effects that are dependent on both RNA hybridisation and catalytic turnover.

(1) An unmodified variant of the classic 10–23 DNAzyme,^55^ “10–23_KRasC[13+12]” (subsequently referred to as “Dz1023”), designed to pair with *KRAS* mRNA residues c.22–c.47 and cleave between c.34 and c.35, dependent on the presence of the c.35G > A [G12D] mutation (Fig. 2(a) and Fig. S1a). When adapting 10–23 to cleave *KRAS* RNA substrates previously,^17^ we found that the binding arms, if unmodified, must be extended beyond their original 7 + 7 or 8 + 8 nt configuration to achieve detectable activity under quasi-physiological (QP) conditions (37 °C, 1 mM Mg^2+^, pH 7.4) on the 2.1 kb “Sub_KRAS_ORF [G12D]” transcript: *k*_obs_ = 0.027 h^−1^ (Fig. 2(a)).

(2) A modified DNAzyme, “10–23_v46”,^18^ designed to pair with *KRAS* mRNA residues c.27–c.41 and cleave between c.34 and c.35, dependent on the presence of the c.35G > U [G12V] mutation (Fig. 2(b) and Fig. S1b). This catalyst is based on the classic 10–23 motif with positions modified with 2′OMe-RNA, 2′MOE-RNA or LNA sugars and PS linkages, identified through chemical mutagenesis^18^ to improve activity in low (≤1 mM) [Mg^2+^]; on the 2.1 kb “Sub_KRAS_ORF [G12V]” transcript: *k*_obs_ = 0.12 h^−1^ under QP conditions (Fig. 2(b)).

(3) An XNAzyme, “FR6_1_KRas12B”^17^ (subsequently referred to as “Fz12B”), fully composed of FANA with terminal PS bonds, designed to pair with *KRAS* mRNA residues c.22–c.47 and cleave between c.33 and c.34, dependent on the presence of the c.35G > A [G12D] mutation (Fig. 2(c) and Fig. S1c). Under QP conditions on the 2.1 kb “Sub_KRAS_ORF [G12D]” transcript *k*_obs_ = 0.23 h^−1^ (Fig. 2(c)).

### Catalysts retain activity following cell lysis treatments

The first step in cellular RNA quantification protocols involves lysis of cells, which can be performed using non- or minimally denaturing buffers (*e.g.* RIPA) or buffers containing the chaotropic denaturant guanidinium thiocyanate (GTC) (*e.g.* TRIzol, Qiagen RLT). Although this would be expected to disrupt hydrogen bonding networks crucial to catalytic function, we reasoned that unlike protein enzymes, catalyst denaturation would likely be reversible. Although no activity was observed in lysis buffer themselves (Fig. S2a) (presumably also due to their lack of metal ion cofactors), we found that all three catalysts indeed carried over and retained their capacity to cleave their short RNA substrates following incubation in GTC-containing TRIzol lysis reagent, followed by phenol extraction and ethanol precipitation, with identical activities to untreated controls (Fig. S2b).

### Catalysts are incompletely degraded by DNase treatments

**Fig. 3:**
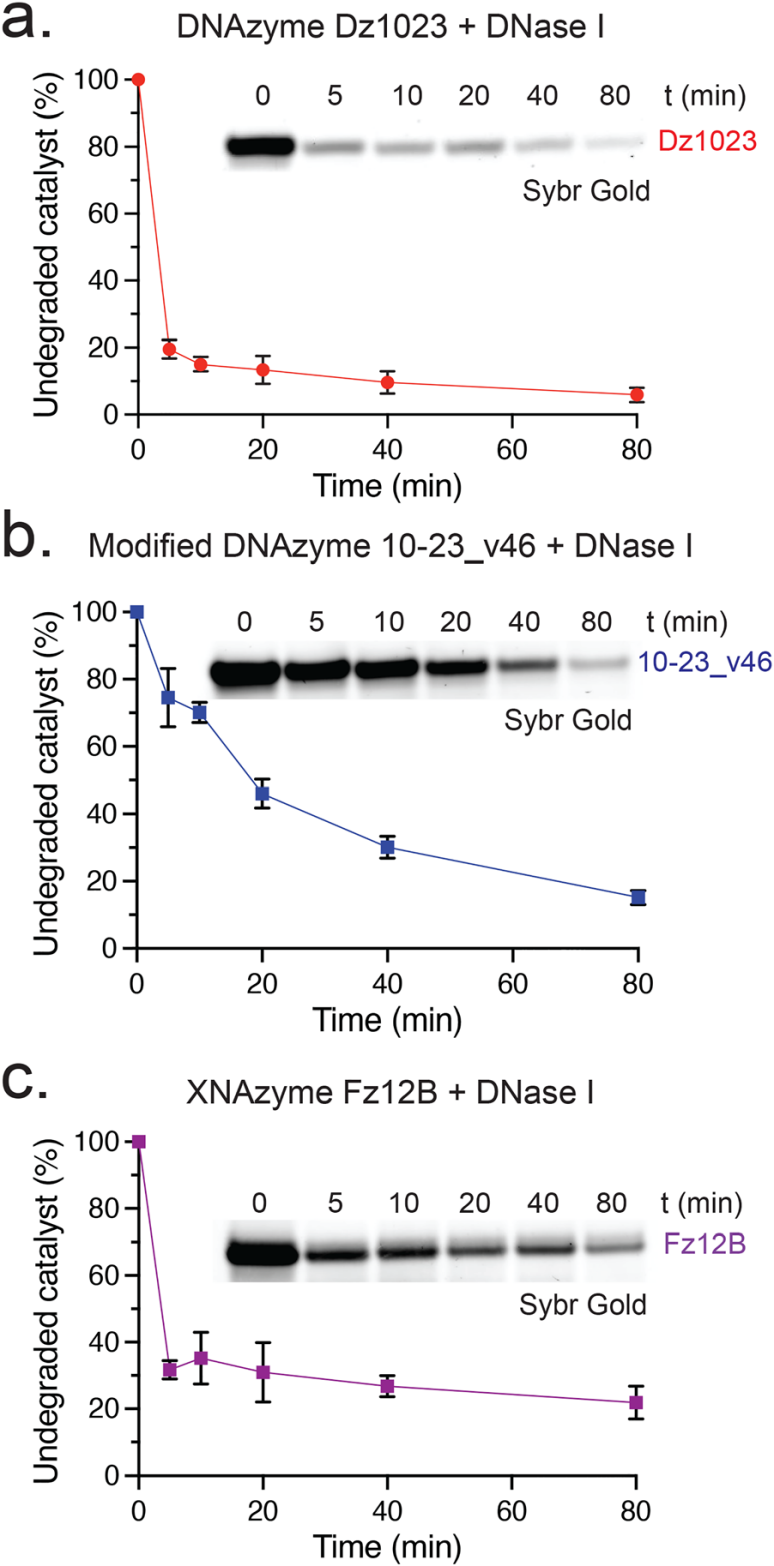
**Typical DNase I treatment does not fully degrade DNAzyme and XNAzyme catalysts, exacerbated by nuclease-resistant modifications**. Graphs and example Urea-PAGE gels stained with Sybr Gold stain to visualise oligonucleotide catalysts (1 μM) treated with DNase I at 37 °C in DNase buffer (10 mM Tris-HCl, pH 7.6, 2.5 mM MgCl_2_, 0.5 mM CaCl) for the times indicated; (a) unmodified DNAzyme Dz1023, (b) modified DNAzyme 10-23_v46, and (c) XNAzyme Fz12B. Data and error bars represent mean ± SEM of three independent experiments.

Following cell lysis and nucleic acid extraction, RNA workflows may incorporate a DNA degradation step; recent examples of oligo catalyst studies involve incubations with 0.1 U μL^−1^ DNase I for 30–60 minutes.^16,18^ As DNase I has a lower activity on single-stranded DNA (particularly if hybridised to RNA) compared with double-stranded DNA,^56,57^ we wondered whether standard protocols may be insufficient to remove catalysts, especially if modified with nuclease-resistant chemistries. Timecourses of unmodified DNAzyme Dz1023, modified DNAzyme 10–23_v46 or XNAzyme Fz12B degradation by DNase I under typical reaction conditions (Fig. 3) revealed that compared with unmodified Dz1023 (Fig. 3(a)), the modified catalysts 10–23_v46 (Fig. 3(b)) and (to a lesser extent) Fz12B (Fig. 3(c)) were degraded ∼5–10-fold slower, suggesting that indeed as much as ∼40% of a modified catalyst could remain after a 30 min treatment (Fig. 3(b)). Notably, ∼10–20% of all three catalysts remained even after extending the incubation to 80 min. We also performed DNase treatments using TURBO DNase (Invitrogen), a variant engineered for higher affinity for DNA (Fig. S3). Although this nuclease exhibited improved activity on 10–23_v46 and Fz12B, degradation was again incomplete, with ∼20% of all three catalysts intact (as judged by Urea-PAGE) after 80 min (Fig. S3). These findings suggest that DNase treatments alone cannot be assumed to have fully inactivated oligo catalysts carried over from cell lysate into RNA preparations, even with extended incubation times.

### Catalysts cleave target RNAs in DNase reaction conditions

**Fig. 4:**
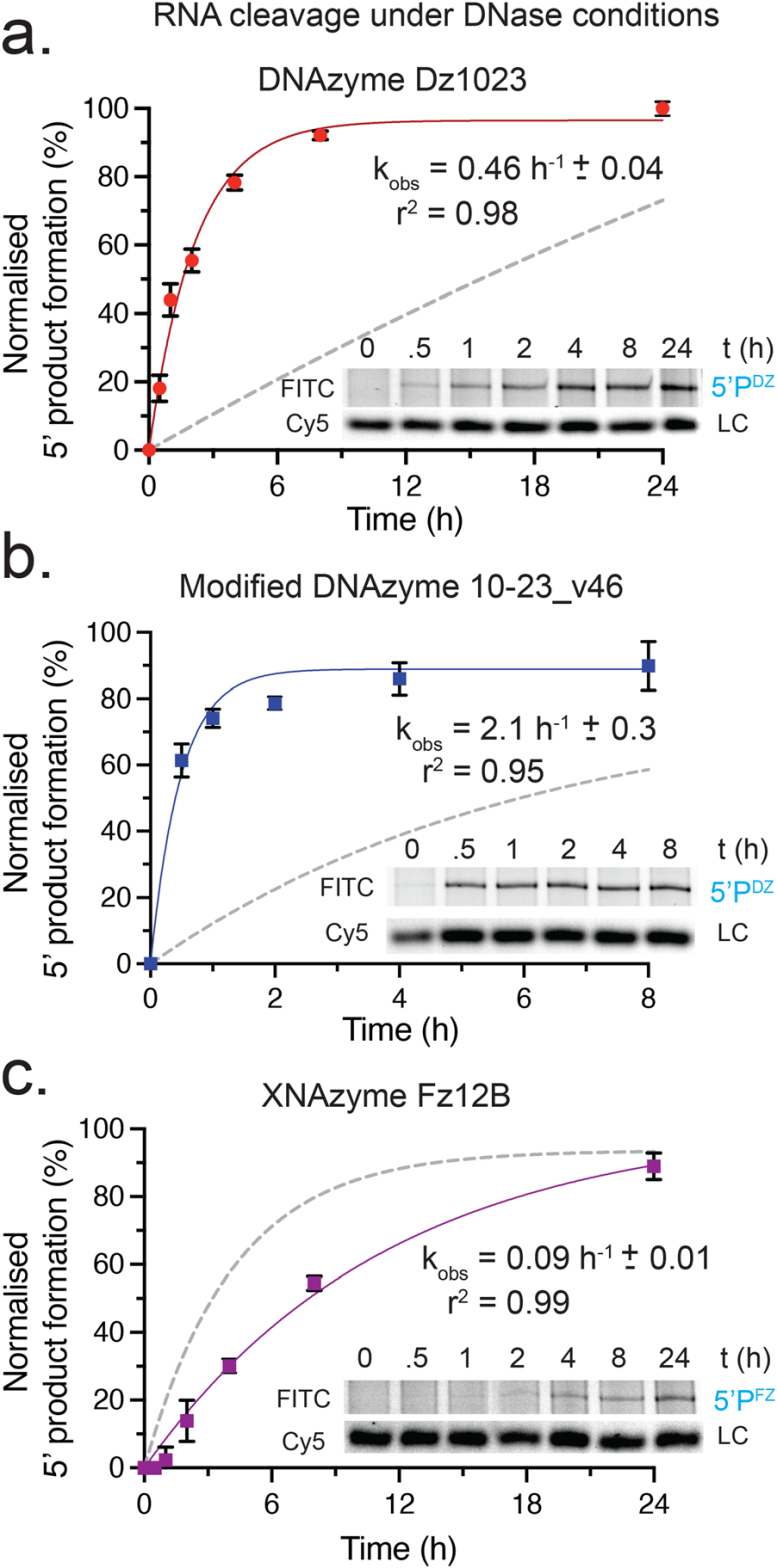
**DNAzyme and XNAzyme catalysts cleave *KRAS* transcripts in typical DNase treatment conditions with comparable or enhanced rates**. Graphs and example Urea-PAGE gels showing timecourses of single-turnover cleavage of FITC-labelled *KRAS* transcripts (1 μM) by each catalyst (5 μM) at 37 °C in DNase buffer (10 mM Tris-HCl, pH 7.6, 2.5 mM MgCl_2_, 0.5 mM CaCl); (a) unmodified DNAzyme Dz1023, (b) modified DNAzyme 10-23_v46, and (c) XNAzyme Fz12B. Data and error bars represent mean ± SEM of 5′ product (5′P) formation normalised to a Cy5-labelled DNA oligo loading control (LC) in three independent experiments, with fit used to calculate apparent rate constant (*k*_obs_). Solid lines show fits used to calculate apparent rate constant (*k*_obs_), dashed lines show fits from reactions in quasi-physiological conditions (as shown in Fig. 2) for comparison.

As typical reaction requirements for DNases (37 °C, pH > 7, divalent metal cofactors^58,59^) overlap those of RNA-cleaving oligo catalysts, we reasoned that DNAzymes or XNAzymes could concomitantly perform RNA cleavage during steps to degrade them. Although such reactions would involve a complex interplay of DNAzymes or XNAzymes acting both as substrates (of DNases) and catalytic binders (of RNA), with *inter alia* partially degraded catalysts presumably exhibiting different cleavage kinetics, for simplicity we sought to determine the effect of DNase I buffer on the activity of the three catalysts in the absence of the nuclease (Fig. 4).

All three catalysts were found to be active in DNase reaction conditions, with the unmodified Dz1023 DNAzyme exhibiting ∼10-fold faster cleavage than in quasi-physiological (QP) conditions (*k*_obs_ = 0.28 h^−1^*vs.* 0.027 h^−1^(QP)) (Fig. 4(a)) and the modified 10–23_v46 ∼17-fold faster cleavage (*k*_obs_ = 2.0 h^−1^*vs.* 0.12 h^−1^ (QP)) (Fig. 4(b)). The XNAzyme Fz12B exhibited a comparable (∼2-fold slower) rate (*k*_obs_ = 0.10 h^−1^*vs.* 0.23 h^−1^ (QP)) (Fig. 4(c)). These results are consistent with the relatively higher concentration of divalent metal cations in DNase I buffer (2.5 mM Mg^2+^, 0.5 mM Ca^2+^) and the higher dependency of 10–23-based DNAzymes on magnesium compared with Fz12B.^17^ We have previously shown that FR6_1-based FANA XNAzymes (from which the Fz12B core is derived) cannot substitute Ca^2+^ for Mg^2+^ in the cleavage reaction,^17^ unlike the 10–23 DNAzyme.^60^ Although long (24 h) timecourses were used to determine rates, these results nevertheless suggest that significant cleavage (up to 70% in the case of 10–23_v46) could occur during a typical 30–60 minute DNase treatment step. Moreover, this would be likely even greater in alternative DNase buffers containing higher concentrations of Mg^2+^ and Ca^2+^ such as Ambion′s “TURBO” DNase buffer.^61,62^

### Catalysts cleave target RNAs in reverse transcription (RT) reaction conditions

Although it is possible to quantify RNA by direct methods (*e.g.* Northern blotting or nanopore sequencing), RNA knockdown is typically determined by reverse transcription (RT) into complementary DNA (cDNA), which is subsequently quantified by real-time PCR (qPCR) or digital PCR (dPCR) assays. RT reactions are typically run at elevated temperatures (42–60 °C), metal cation concentrations (1.5–3 mM Mg^2+^) and pH 8.3, which we reasoned would also be favourable for RNA-cleavage by carried over DNAzymes or XNAzymes.

**Fig. 5:**
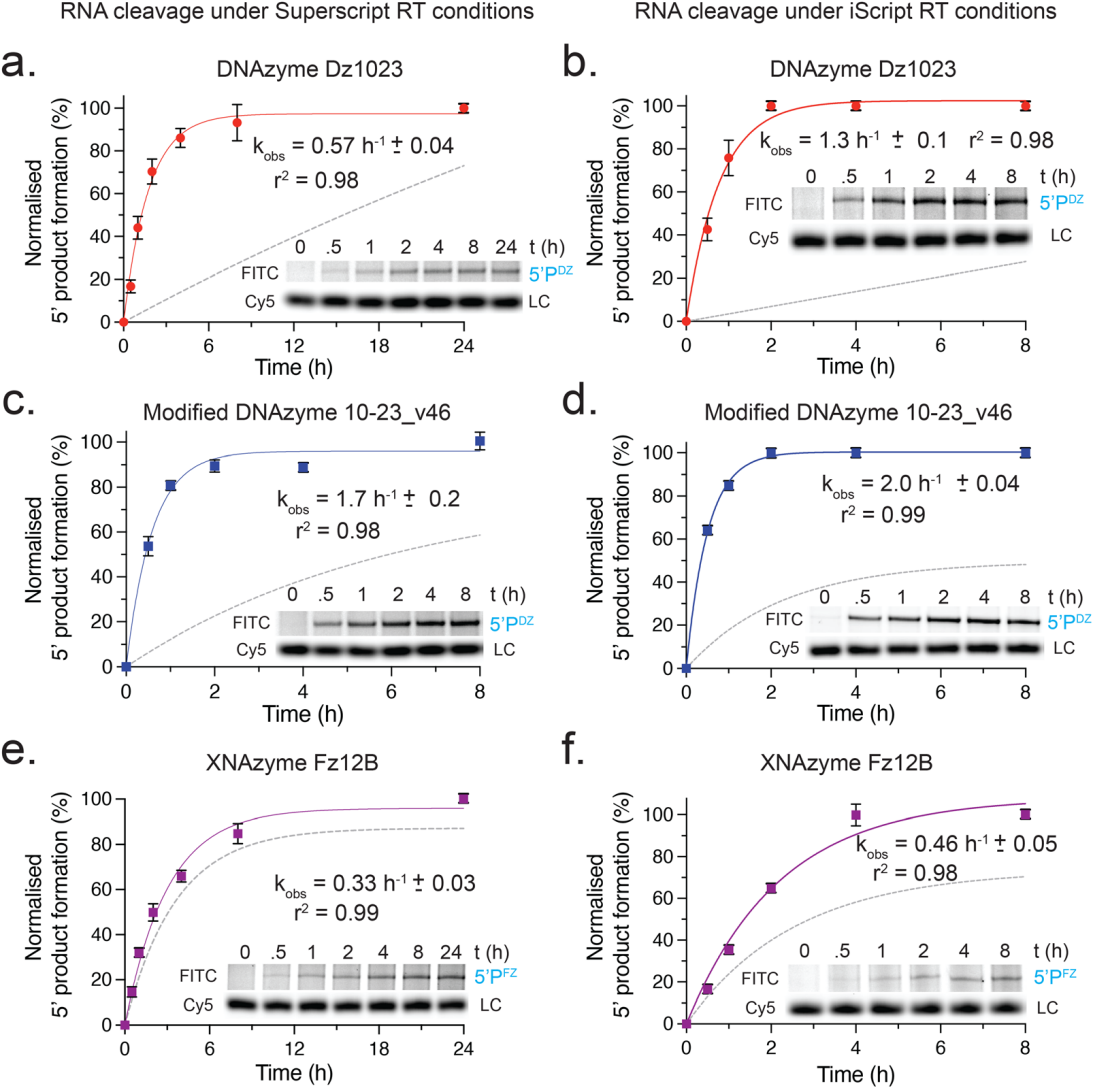
**DNAzyme and XNAzyme catalysts cleave *KRAS* transcripts in typical reverse transcription (RT) conditions with comparable or enhanced rates**. Graphs and example Urea-PAGE gels showing timecourses of single-turnover cleavage of FITC-labelled *KRAS* transcripts (1 μM) by each catalyst (5 μM) at (a, c, e) 50 °C in 50 mM Tris-HCl, pH 8.3, 75 mM KCl, 3 mM MgCl_2_, or at (b,d,f) 46 °C in iScript buffer; (a, b) unmodified DNAzyme Dz1023, (c, d) modified DNAzyme 10-23_v46, and (e, f) XNAzyme Fz12B. Data and error bars represent mean ± SEM of 5′ product (5′P) formation normalised to a Cy5-labelled DNA oligo loading control (LC) in three independent experiments. Solid lines show fits used to calculate apparent rate constant (*k*_obs_), dashed lines show fits from reactions in quasi-physiological conditions (as shown in Fig. 2) for comparison.

To evaluate the general potential for cleavage activity during such steps, we determined the rate constants (*k*_obs_) of the three catalysts cleaving their synthetic transcripts in two commonly used commercial RT reaction conditions (for simplicity, in the absence of polymerase) (Fig. 5): (1) SS: superscript buffer (Thermo Fisher Scientific) at 50 °C, typically used for RT reactions prior to qPCR, and (2) iS: iScript buffer (Bio-Rad) at 46 °C, typically used for RT reactions prior to dPCR.

All three catalysts were found to be active in the RT conditions. The cleavage rates of unmodified DNAzyme Dz1023 was found to be enhanced by ∼20-fold under the Superscript conditions (0.63 h^−1^ (SS) *vs.* 0.027 h^−1^ (QP)) (Fig. 5(a)) and ∼60-fold under the iScript conditions (1.6 h^−1^ (iS) *vs.* 0.027 h^−1^ (QP)) (Fig. 5(b)). Likewise, activity of the modified 10–23_v46 DNAzyme was enhanced in both RT conditions, by ∼17-fold (2.0 h^−1^ (SS) and 2.04 h^−1^ (iS) *vs.* 0.12 h^−1^ (QP)) (Fig. 5(c) and (d)). The Fz12B XNAzyme, however, exhibited comparable rates in both RT conditions (0.32 h^−1^ (SS) and 0.36 h^−1^ (iS) *vs.* 0.23 h^−1^ (QP)) (Fig. 5(e) and (f)). These findings indicate that any residual oligo catalyst from earlier steps will continue to be active and could affect substantial cleavage of target transcripts (40–80% in the case of the two DNAzymes) in a typical 30–60 minute RT reaction.

### Catalysts can inhibit reverse transcription of target RNA

**Fig. 6:**
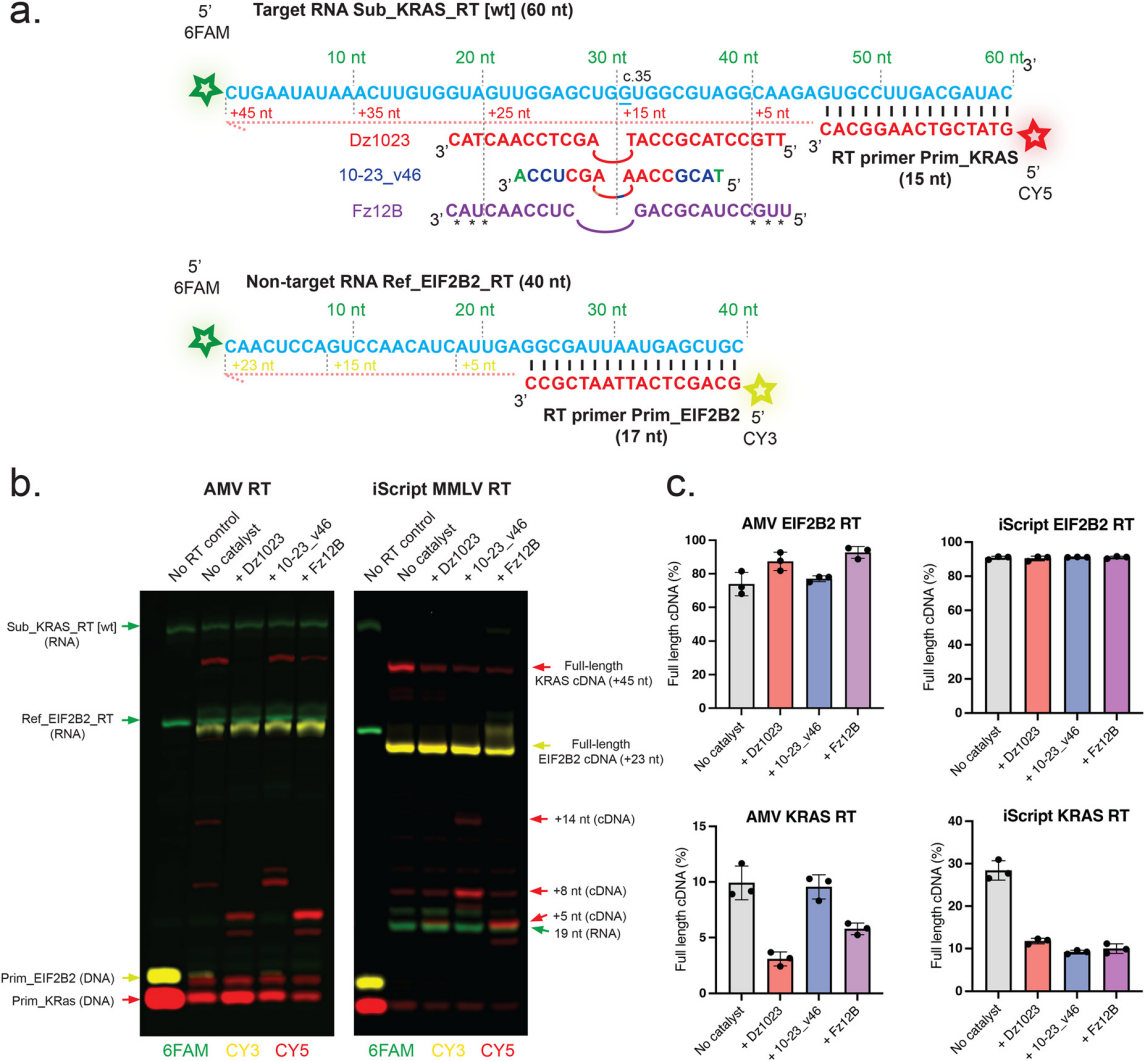
**DNAzyme and XNAzyme catalysts can inhibit RT polymerase processivity and trigger their RNase H activity at their binding sites**. (a) RNA and oligonucleotide catalyst sequences, (b) composite of images of Urea-PAGE gels and (c) bar charts of quantified full-length cDNA in one-pot RNA RT assays comprising (0.1 μM) *KRAS* and *EIF2B2* short RNA templates and (0.1 μM) template-specific primers 5′-labelled with fluorophores as indicated, and either AMV or iScript (MMLV) RT polymerase, using their respective manufacturers’ recommended conditions (see Materials and Methods; AMV (1 h, 42 °C; iScript 5 min at 25 °C, 20 min at 46 °C). Reactions were spiked with (0.1 μM) either unmodified DNAzyme Dz1023, modified DNAzyme 10-23_v46, XNAzyme Fz12B or buffer alone. Note that although the Sub_KRAS_RT RNA comprises the catalysts’ target site, the wild-type sequence was used (*KRAS* c.35G), so catalysts will bind but have little to no intrinsic cleavage activity in order to exclude this from the assay. Bars and errors represent mean full-length cDNA as a percentage of extended primer ± SEM in three independent experiments. Individual gel images comprising the composite are shown in Fig. S4.

In addition to potential RNA cleavage during the RT reaction, we also wondered whether mismeasurements could arise from catalytic oligos affecting the activity of reverse transcriptases. To investigate this, we developed a one-pot reverse transcription primer extension assay comprising two template and primer pairs for cDNA synthesis (Fig. 6(a)): (1) a 60 nt 6FAM-labelled *KRAS* wild-type RNA (“Sub_KRAS_RT [wt]”), which contains the binding sequence for the three catalysts but not the G12D or G12V mutations necessary for DNAzyme- or XNAzyme-mediated cleavage (*i.e.* enabling catalysts to hybridise, but preventing or broadly limiting their catalytic turnover, for simplicity) and a Cy5-labelled DNA primer (“Prim_KRAS”), and (2) a 40 nt 6FAM-labelled *EIF2B2* non-targeted RNA (“Ref_EIF2B2_RT”) and a Cy3-labelled DNA primer (“Prim_EIF2B2”). The assay was designed to differentiate possible effects dependent on RNA binding (which would affect the RT of (1) but not (2)) and binding-independent or non-sequence specific effects (which would affect the RT of both (1) and (2)).

We performed RT reactions, spiked with catalysts, using three typical RT polymerases (RTpols) used in qPCR – AMV RTpol and engineered MMLV RTpol Superscript III – or dPCR – iScript (Bio-Rad), also an engineered MMLV RTpol – and used Urea-PAGE to assess full-length and prematurely terminated cDNA products (Fig. 6(b) and Fig. S4). In all combinations of catalysts and RTpols, the non-targeted *EIF2B2* RNA was transcribed with comparable efficiently as no-catalyst controls (60–90% full-length cDNA) (Fig. S4). This suggests that the catalysts had no general sequence-independent inhibitory effect, although it appears that the *EIF2B2* RNA was a less challenging template than the *KRAS* RNA (which yielded ∼4-fold lower proportion of full-length cDNA in all no-catalyst controls) (Fig. S4), so small reductions in RTpol activity may be less visible. In contrast, all three catalysts were found to significantly inhibit the *KRAS* RNA RT when spiked into reactions with iScript RTpol (∼65% reduction in full-length cDNA), and likewise the unmodified Dz1023 and XNAzyme Fz12B (but not 10–23_v46) when spiked into reactions with AMV RTpol (75% and 50% reduction in full-length cDNA, respectively) (Fig. 6(b), (c) and Fig. S4a, b). Superscript III RTpol was seemingly less affected by any of the catalysts (Fig. S4c).

The electrophoretic mobilities of short cDNAs observable in catalyst-spiked RT reactions were consistent with RTpols terminating at positions in the *KRAS* template predicted to hybridise with the binding arms of each catalyst (Fig. 6(a), (b) and Fig. S4a, b). We reasoned that this could derive from either from steric blocking of RTpol read-through (*i.e.* failure of their strand displacement activity) and/or by induction of RTpol RNase H activity (which was also evident from the appearance of short RNAs, most prominently with iScript RTpol) (Fig. 6(b) and Fig. S4b). Catalysts (depending on their backbone chemistries) hybridised to RNAs can function as substrates for RNase H enzymes; AMV RTpol and iScript RTpol possess RNase H activity, Superscript III has been engineered to have little to no RNase H activity. To rule out the possibility that the higher temperature of the Superscript III RT reactions (50 °C) compared with the AMV (46 °C) and iScript (42 °C) RTs simply melts the catalyst:RNA heteroduplex and prevents either effect, we also performed reactions at 42 °C and observed comparable results to those at 50 °C (Fig. S4d).

To differentiate between inhibition of RTpol processivity and induction of RTpol RNase H activity, we compared RT reactions (using iScript RTpol) with or without the DNA primers (Fig. S5a). These reactions revealed that both the unmodified DNAzyme Dz1023 and (to a lesser degree) the modified DNAzyme 10–23_v46 were indeed able to induce RTpol RNase H-mediated cleavage of the *KRAS* RNA, whereas the FANAzyme Fz12B was not (Fig. S5a) and may even partially inhibit this activity in an non-sequence-specific manner (Fig. S4b and Fig. 6(b)). This is consistent with previous results showing all-FANA antisense oligos are worse substrates of RNase H than DNA or mixed DNA-FANA equivalents.^63^ Broadly, these results suggest that (aside from intrinsic catalyst-dependent cleavage of the RNA template, which was excluded from this assay) both steric hindrance of the RTpol as well as RTpol RNase H activity can contribute to catalyst-induced reduction in target site cDNA synthesis.

### DNAzymes can function as primers for cDNA synthesis during RT reactions

In the absence of RT primers, we were surprised to observe that Dz1023 and 10–23_v46 were apparently able to induce the cleavage of the 60 nt *KRAS* RNA by the RNase H activity of iScript RTpol (Fig. S5a) at sites 4–9 nt away from the hybridised catalysts (position 13 in Sub_KRAS_RT) (Fig. S5b). We hypothesised that the 3′ binding arm of Dz1023 (being unmodified DNA) may function as a primer for RT, enabling cDNA synthesis and thus generating heteroduplex substrates for RTpol RNase H activity. Although the 3′ terminal residue of the modified 10–23_v46 DNAzyme is an LNA-A, MMLV RTpols have been shown to possess some capacity to extend LNA residues;^64^ templated extension of 10–23_v46 to generate cDNA would also explain it′s apparent ability to induce RTpol RNase H activity (Fig. S5a) despite modification of binding arms with LNA and 2′OMe-RNA, analogues that do not trigger RNase H.^65^

**Fig. 7:**
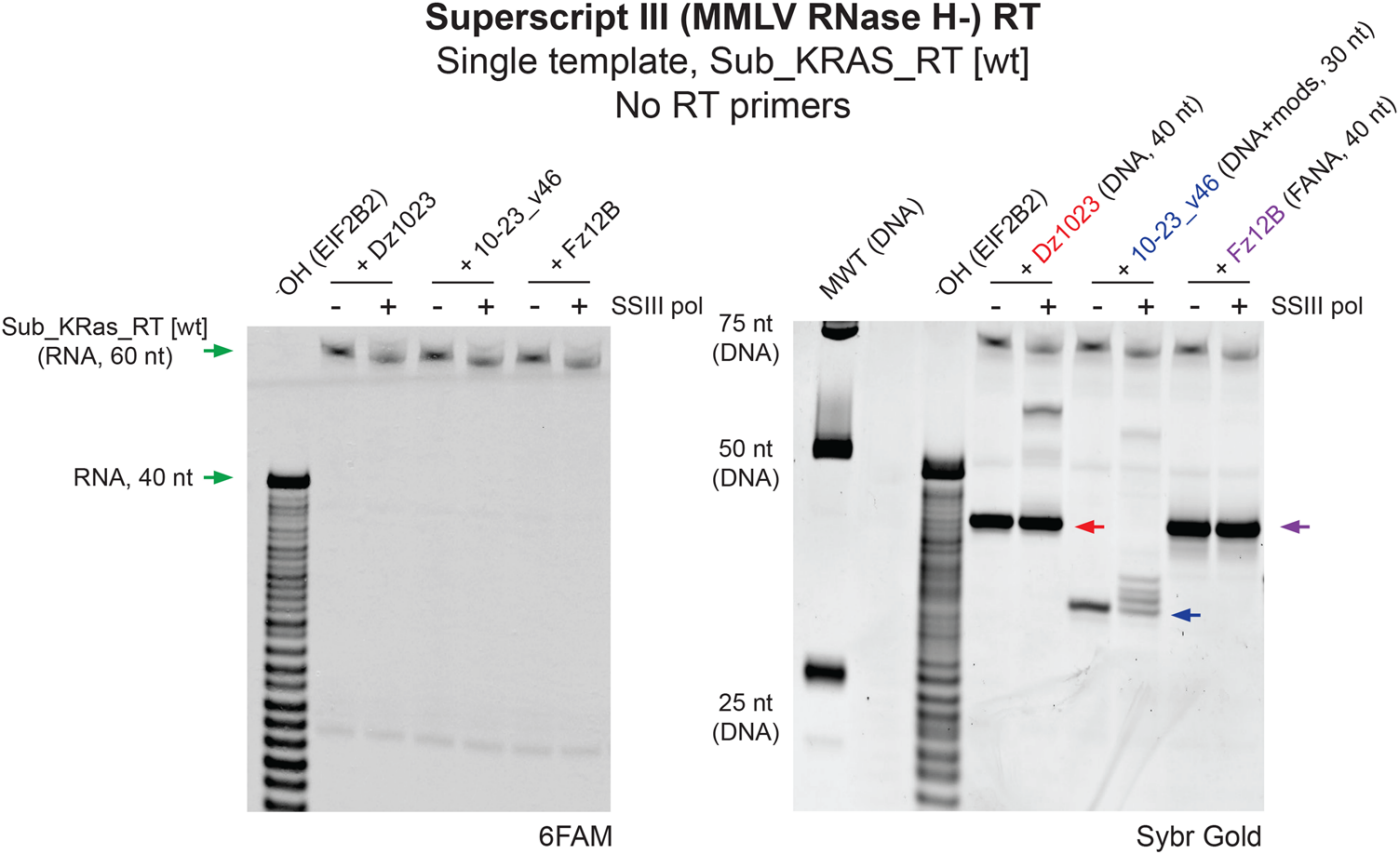
**DNAzyme catalysts can act as primers during RT reactions**. Urea-PAGE gel (imaged for 6FAM fluorescence or subsequently stained and imaged for all nucleic acids using Sybr Gold stain, as indicated) showing short RNA reverse transcription assays comprising 0.1 μM 6FAM-labelled RNA template Sub_KRAS_RT [wt] (60 nt) without template-specific primers, and spiked with either 0.1 μM unmodified DNAzyme Dz1023, modified DNAzyme 10-23_v46 or XNAzyme Fz12B, with or without Superscript III (MMLV; RNase H-) RT polymerase. (-OH) indicates partially alkaline hydrolysed RNA used as a molecular weight marker.

To test whether the catalysts could function as primers during an RT reaction, we repeated the short RNA RT control assays (this time in the absence of the DNA RT primers and using Superscript III RTpol to exclude RNase H activity) and analysed them by Urea-PAGE gel stained with SYBR Gold (Invitrogen) to visualise the unlabelled catalysts (Fig. 7). This revealed RTpol-dependent electrophoretic mobility shifts consistent with RNA-templated 3′ extension of both Dz1023 and 10–23v46, but not Fz12B (whose 3′ terminal is FANA) (Fig. 7).

To further confirm this, we modified the 3′ terminal residue of Dz1023 with a chain terminator, 2′,3′-dideoxy ribose (“Dz1023_ddC”), which did not affect the DNAzyme′s catalytic function (Fig. S6a) but indeed eliminated extension of the DNAzyme during the RT reaction (Fig. S6b). Consistent with the prevention of DNAzyme priming, when similar no-primer RT reactions were performed using iScript RTpol (*i.e.* reintroducing RNase H activity), the apparent DNAzyme-dependent RTpol-mediated cleavage of the 60 nt *KRAS* RNA at position 13 was also eliminated (Fig. S6c). However, RTpol-dependent specific cleavage of the full-length *KRAS* RNA was still observed upon addition of Dz1023_ddC (Fig. S6c and d), with a concurrent appearance of 35 and 38 nt RNA fragments (Fig. S6c and d), suggesting RTpol RNase H activity is also recruited by hybridised DNAzymes’ binding arms (Fig. S6e). The binding arms of Dz1023_ddC would be expected to yield 12 bp and 13 bp RNase H substrates, which are each shorter than the preferred 14–20 bp substrate of MMLV RTpol^66^ (as we observe in primed RT reactions with iScript RTpol in the absence of catalysts (Fig. 6(b) and Fig. S4b, S6d)). However, we have previously found^36^ that residues in the catalytic core of a DNAzyme, in addition to binding arms, can promote alternative RNA hybridisation modes and contribute to triggering of *E. coli* RNase H and human RNase H1, so presumably also have the capacity to induce RNase H activity of RTpols.

### DNAzymes can affect RNA quantification by acting as RT primers

Next, we sought to explore catalyst-induced mismeasurements in an RT-PCR workflow designed to quantify the more realistic 2.1 kb synthetic *KRAS* transcripts. In these experiments, catalysts were added to target transcripts that encode the G12D or G12V alleles and can thus be bound and cleaved by the catalysts – however, samples were processed immediately following spiking so that, in principle, no cleavage ought to have occurred prior to the RT reaction (*t* = 0 h). For simplicity, DNase treatments were not performed to exclude the possibility of cleavage during this step. cDNA products of RT reactions with iScript RTpol (primed using random hexamers) were quantified using a multiplexed probe-based ddPCR assay that we previously developed to measure cleavage of *KRAS* transcripts.^17^ Briefly, this assay comprises two ‘TaqMan’ primer and probe sets that specifically quantify: (1) the *KRAS* exon 2 site recognised by the catalysts and (2) a downstream site at the *KRAS* exon 3/4 junction. The proportion of the ‘target’ and ‘non-target’ amplicons, double-referenced to no-catalyst controls (to normalise differences in random priming and processivity of RTpols across the transcript), gives the apparent cleavage of the *KRAS* transcript at the exon 2 site. We have previously validated this assay by comparing samples of the same DNAzyme- and XNAzyme-cleavage reactions by both ddPCR and Urea-PAGE.^17^

**Fig. 8:**
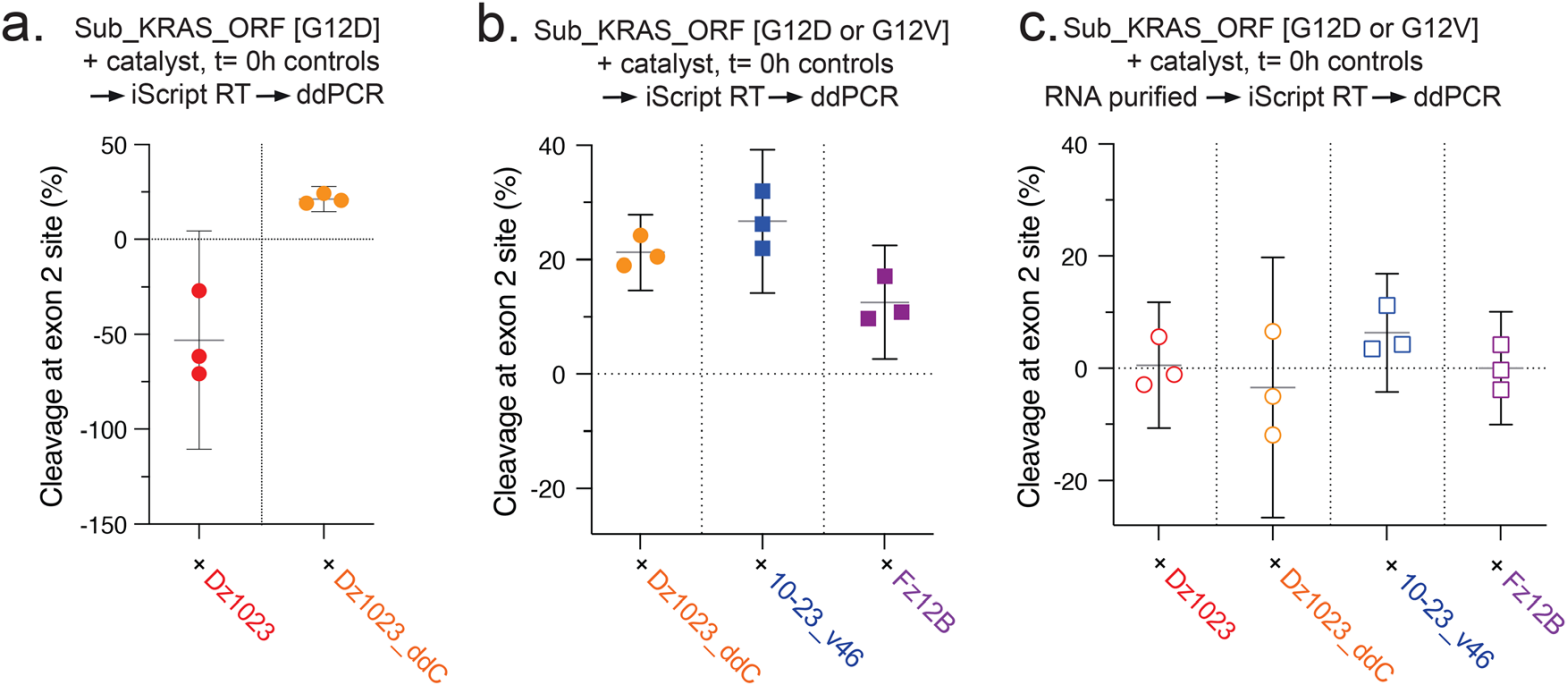
**Oligonucleotide catalysts are capable of false negative and false positive mismeasurements of *KRAS* transcript cleavage by RT-qPCR**. Grouped scatter graphs showing droplet digital quantitative PCR (ddPCR) measurements of apparent cleavage of Sub_KRAS_ORF [G12D] mutant *KRAS* transcripts (1 μM) at the exon 1/2 catalyst target region (normalised to a non-target region, exon 3/4) occurring during reverse transcription reactions (5 min at 25 °C, 20 min at 46 °C) performed with iScript RT polymerase, following spiking with (5 μM) (a) unmodified DNAzyme Dz1023 or a variant with terminal 2′,3′-dideoxy C, “Dz1023_ddC”, or (b, c) modified DNAzyme 10-23_v46 or XNAzyme Fz12B, and performed (b) before or (c) after purification of RNA.

In *KRAS* transcript samples spiked with the unmodified Dz1023 and used directly as templates for RT (*i.e.* unpurified), we were surprised to observe a striking increase in the quantity of the exon 2 site cDNA compared with no-catalyst controls, producing an apparent false negative mismeasurement of cleavage (−54%) (Fig. 8(a)). No-template controls spiked with catalysts yielded no amplicons following RT-ddPCR (Fig. S7a), ruling out detection of the catalysts themselves. When Dz1023 was added after RT, mismeasurement was not observed (Fig. S7b), suggesting the DNAzyme does not affect the PCR step. RNA purification to deplete Dz1023 following spiking but prior to RT (see Materials and Methods) likewise eliminated the effect (Fig. S7b). Given our earlier observation that DNAzymes can undergo RNA-templated 3′ extension by RTpols (Fig. 7 and Fig. S6b, c), we reasoned that the effect may derive from DNAzymes priming reverse transcription and/or enhancing RTpol read-through of RNA secondary structure at the exon 2 binding site. Consistent with DNAzymes acting as primers, the dideoxy 3′-blocked DNAzyme Dz1023_ddC did not produce the false negative effect (Fig. 8(a)).

Cloning and sequencing of the amplicon produced by the ‘Taqman’ primer set used to quantitate the *KRAS* cleavage site RNA revealed a 109 bp sequence spanning the *KRAS* mRNA 5′ UTR to residue c.35 in the ORF (Fig. S8). This was somewhat surprising as the 5′ cleavage product produced by Dz1023 (Fig. 2) therefore contains all but the final 3′ nucleotide of the sequence detected by this assay. The assay nevertheless produces fewer amplicons when RNA is cleaved (by Dz1023_ddC and purified to remove DNAzyme prior to RT) (Fig. S9), presumably as random hexamers used in the RT are insufficient to generate the ‘complete’ 3′ end of the cut RNA. Overall, this suggests that when DNAzymes are present in the RT reaction, their 3′ binding arms can indeed prime and generate cDNA products detectable by this assay – whether cleavage has occurred or not.

Finally, consistent with our observations using the short RNA RT assays, without purification, spiking of Dz1023_ddC, 10–23_v46 or Fz12B into transcript samples resulted in apparent knockdown in the RT-dPCR assay with all three catalysts, to ∼22%, ∼27% and ∼15%, respectively (Fig. 8b). This is consistent with both catalyst-dependent cleavage of transcripts in RT conditions (Fig. 5(b), (d) and (f)) as well as the inhibitory effects on RTpol (Fig. 6(b), (c) and Fig. S4b) and induction of RNase H activity we observed using the 60 nt KRAS RNA (Fig. S5a and S6c, d), but does not include the additional cleavage that could occur during DNase treatment (Fig. 4). As with Dz1023, purification of RNA spiked with the modified DNAzyme 10–23_v46 and XNAzyme Fz12B prior to RT broadly mitigated the apparent knockdown (Fig. 8(c)).

## Discussion

Rigorously studying intracellular activities of nucleic acid catalysts presents a range of underappreciated technical challenges. Here, we sought to explore sources of misleading results that can occur if they are carried over into cellular nucleic acid preparations and assay workflows due to hybridisation with target (and/or off-target) RNA and/or shared physicochemical properties. We have previously found that catalysts can be active in cell lysate prepared using non-denaturing methods,^44^ but find that even when denaturing buffers are used, loss of catalytic activity (presumably due to unfolding of the active conformation) is reversible, enabling RNA cleavage to be catalysed in subsequent assay steps. Counterintuitively, the classic cell lysis denaturant guanidinium thiocyanate has been reported to strengthen RNA hybridisation,^67^ so may enhance catalyst carry over. RNA cleanup protocols may enable depletion of DNAzymes and XNAzymes (as we previously established in our studies of the FANAzyme Fz12B,^17^ and further elaborate here), however few assay workflows reported in the literature involve explicit catalyst removal steps other than DNase treatment, and several appear to omit this step.^28,34,41,68^ However, as we show here, standard DNase treatments may fail to fully degrade DNAzymes, in particular when catalysts are modified with nuclease-resistant analogue chemistries,^16,18,28,30,35,41,69^ or appended to motifs^70^ or nanostructures^71^ designed to sterically hinder exonuclease access to oligo termini. Furthermore, catalysts may avoid cleavage when hybridised to RNA as DNase I and related variants exhibit reduced activity against RNA:DNA heteroduplexes.^57^

Crucially, we find that catalysts (in particular the 10–23 DNAzyme variants we examined) demonstrate markedly enhanced activities (up to ∼60-fold faster) in conditions used in typical RNA assay workflows compared to their potential intracellular activity. Our results suggest that cleavage at any of the three stages in a typical cellular RNA assay workflow (in lysate, during DNase treatment or in a reverse transcription reaction) would be incorrectly interpreted as intracellular knockdown – *i.e.* false positives. Such post-lysis cleavage is challenging to control for as cleavage is dependent on catalysts binding to RNA as well as activity of their catalytic core motifs (on the ‘correct’ RNA target sequence; here we used allele-selective catalysts that cleave *KRAS* G12D or G12V and not wild-type RNA), so would not be accounted for by comparisons with non-binding or catalytically dead DNAzyme or XNAzyme variants.

In addition to these sources of mismeasurement, we uncovered two further mechanisms that could contribute to errors in RNA knockdown assessment using RT-PCR methods. We find that interactions between RT polymerases and catalysts hybridised to RNA templates can cause either: (1) an overestimation of RNA cleavage (*i.e.* another source of false positive results) due to induction of RTpol RNase H activity and/or steric hindrance of cDNA synthesis, or (2) an underestimation of RNA cleavage due to amplification of target site cDNA (*i.e.* a source of false negative results). Neither of these effects is dependent on the intrinsic turnover of oligo catalysts themselves (so may be less specific) but would likely be affected by the length and chemistry of their binding arms. If catalysts are tightly bound to RNAs, RTpols may lack sufficient strand displacement activity to prevent stalling, whereas if RTpols possess RNase H activity, they may recognise the heteroduplex formed by RNA-bound catalysts as substrates for cleavage – in both cases limiting target site cDNA synthesis. However, if the 3′ binding arm of a catalyst can be processed by RTpol as a primer-template duplex, catalysts may be subject to template-dependent extension and the resulting catalyst-cDNA chimeras subsequently detected as target site cDNA (depending on PCR primers and probes). In the case of the unmodified Dz1023 DNAzyme, the latter false negative effect appeared to dominate over false positive effects in our RT-dPCR assay.

Beyond the sources of mismeasurement identified in our study, the development of faster catalysts with substantially reduced requirement for metal cofactors, including magnesium-independent DNAzymes,^72^ suggests that it will become increasingly challenging to exclude the possibility of cleavage during sample handling or storage steps. Nucleic acid catalysts have been reported^41^ to be capable of chemical transformations in water ice within the temperature range found in laboratory freezers^73^ suggesting that false positives could arise due to cleavage in the liquid eutectic phase that can form in frozen samples. Our results suggest caution must be taken in interpreting DNAzyme and XNAzyme intracellular experiments and that, where possible, control samples should be taken immediately following transfection or uptake of catalysts into cells (*i.e.* a zero time point) as standard practice to evaluate possible post-lysis effects. Our study also highlights that RNA purification (following lysis in conditions in which catalysts are not active) to prevent catalyst carry over offers a viable strategy for mitigating the sources of mismeasurement we describe, although protocols may have to be developed to accommodate the properties of novel catalysts, *e.g.* with enhanced affinity for RNA. We hope that improved understanding of DNAzyme and XNAzyme intracellular activities – as well as those of other RNA targeting systems such as oligonucleotide-conjugated chemical nucleases,^74^ whose intracellular effects^75,76^ may also be confounded by the sources of mismeasurement we describe, will further enhance the prospects for applications of these promising technologies as precision RNA tools.

## Author contributions

A. I. T. conceived and directed the project, together with M. J. D. M. J. D. and A. I. T. acquired, curated and analysed data, mass spectrometry was performed by A. M. A. I. T. wrote the manuscript with contributions from the other authors.

## Conflicts of interest

There are no conflicts to declare.

## Supplementary Material

CB-006-D5CB00182J-s001

## Data Availability

The data supporting this article have been included as part of the SI. Supplementary Fig. 1–9. Oligonucleotide sequences are provided in supplementary Table 1. See DOI: https://doi.org/10.1039/d5cb00182j.
