# Supplementary material for: Sources of mismeasurement of RNA knockdown by DNAzymes and XNAzymes
Source: RSC Chem Biol. 2025 Sep 8;6(10):1595–606. doi: 10.1039/d5cb00182j (PMC12426771; doi:10.1039/d5cb00182j)
Supplement: CB-006-D5CB00182J-s001 [file CB-006-D5CB00182J-s001.pdf]

Supplementary Information (SI) for RSC Chemical Biology.
This journal is © The Royal Society of Chemistry 2025

Supplementary Material for:

Sources of mismeasurement of RNA knockdown by DNAzymes and XNAzymes

Maria J. Donde[a,b], Alicia Montulet[a], Alexander I. Taylor*[a,b]

[a] Department of Chemistry, King's College London, Britannia House, 7 Trinity street, London SE1 1DB, United Kingdom

[b] Cambridge Institute of Therapeutic Immunology & Infectious Disease (CITIID), University of Cambridge, Cambridge Biomedical Campus, Cambridge CB2 0AW, United Kingdom

*corresponding author: alex.taylor@kcl.ac.uk

Contents:

Supplementary Figures 1 – 9.

Supplementary Table 1.

Materials and Methods.

Supplementary References.

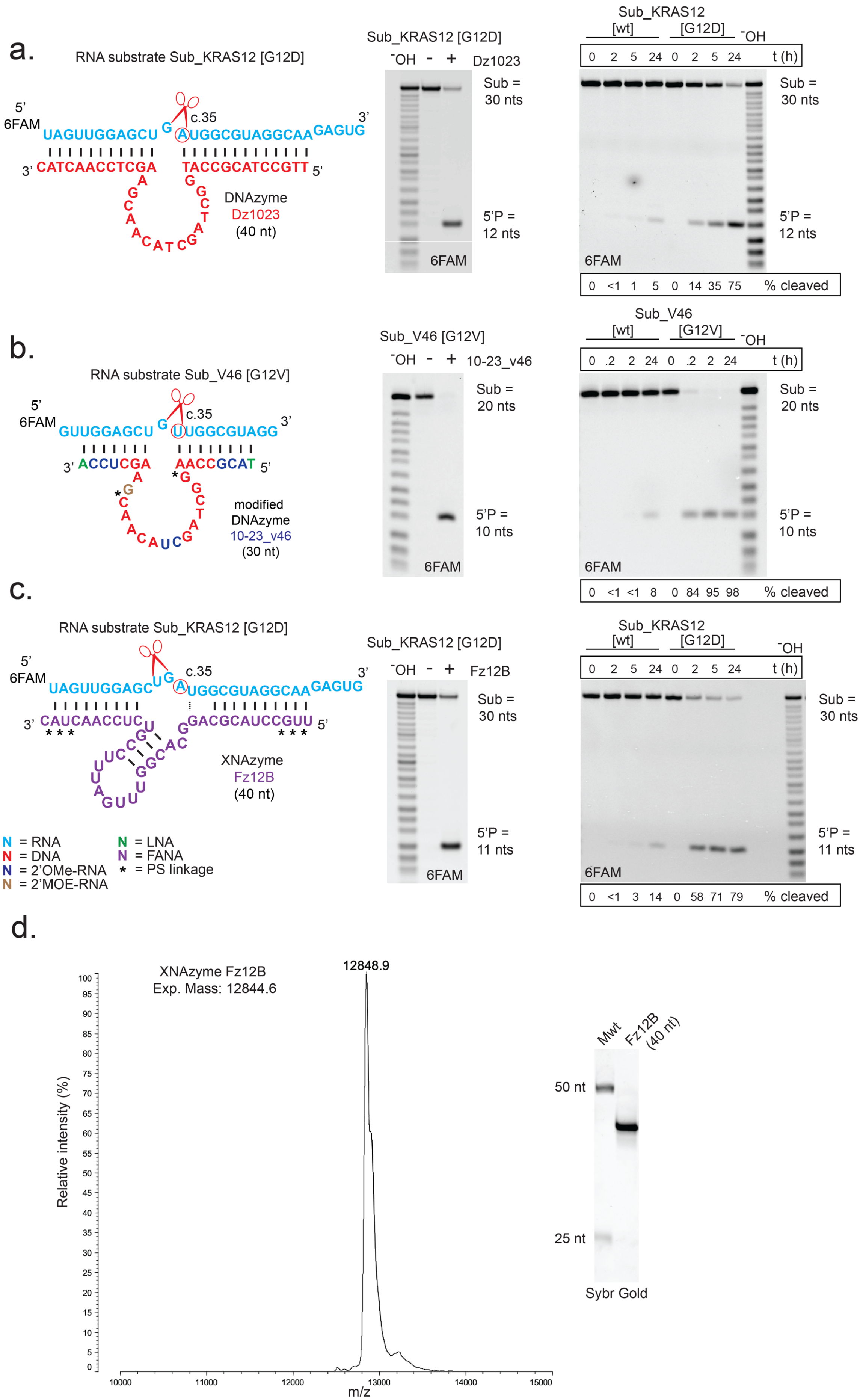
a.
RNA substrate Sub_KRAS12 [G12D]
5' 6FAM
UAGUUGGAGCU G A UGGCGUAGGCAA GAGUG 3'
c.35
3' CATCAACCTCGA TACCGCATCCGTT 5'
DNAzyme
Dz1023
(40 nt)
Sub_KRAS12 [G12D]
-OH - + Dz1023
Sub = 30 nts
5'P = 12 nts
6FAM
Sub_KRAS12
[wt] [G12D] -OH
0 2 5 24 0 2 5 24 t (h)
0 <1 1 5 0 14 35 75 % cleaved
b.
RNA substrate Sub_V46 [G12V]
5' 6FAM
GUUGGAGCU G UUGGCGUAGG 3'
c.35
3' ACCUCGA AACCGCAT 5'
modified
DNAzyme
10-23_v46
(30 nt)
Sub_V46 [G12V]
-OH - + 10-23_v46
Sub = 20 nts
5'P = 10 nts
6FAM
Sub_V46
[wt] [G12V] -OH
0 .2 2 24 0 .2 2 24 t (h)
0 <1 <1 8 0 84 95 98 % cleaved
c.
RNA substrate Sub_KRAS12 [G12D]
5' 6FAM
UAGUUGGAGC U G A UGGCGUAGGCAA GAGUG 3'
c.35
3' CAUCAACCUC GACGCAUCCGUU 5'
XNAzyme
Fz12B
(40 nt)
N = RNA
N = DNA
N = 2'OMe-RNA
N = 2'MOE-RNA
N = LNA
N = FANA
* = PS linkage
Sub_KRAS12 [G12D]
-OH - + Fz12B
Sub = 30 nts
5'P = 11 nts
6FAM
Sub_KRAS12
[wt] [G12D] -OH
0 2 5 24 0 2 5 24 t (h)
0 <1 3 14 0 58 71 79 % cleaved
d.
XNAzyme Fz12B
Exp. Mass: 12844.6
12848.9
Relative intensity (%)
m/z
Mwt
Fz12B (40 nt)
50 nt
25 nt
Sybr Gold

Supplementary Figure 1. DNAzyme and XNAzyme oligonucleotide catalysts capable of selective cleavage of KRAS RNA sequences encoding disease-associated codon 12 mutants.

*RNA-cleaving catalysts and their short KRAS RNA substrates used in previous studies; (**a**) unmodified DNAzyme Dz1023*[1, 2]*, (**b**) modified DNAzyme 10-23_v46*[3]*, and (**c**) XNAzyme Fz12B*[2]*. Red scissors indicate site of cleavage and red circles indicates RNA base (equivalent to KRAS c.35) that determines selectivity of cleavage between wild-type (c.35G) and mutant sequences as shown. Urea-PAGE gels (centre) show examples of single-turnover cleavage of 6FAM-labelled short KRAS RNAs (1 μM) by each catalyst (5 μM) and (right) reactions with wild-type (c.35G) or mutant (c.35A; G12D, or c.35U; G12V) substrates for the times indicated. (d) MALDI-ToF mass spectrum and Urea-PAGE gel showing in house synthesised FANA XNAzyme Fz12B.*

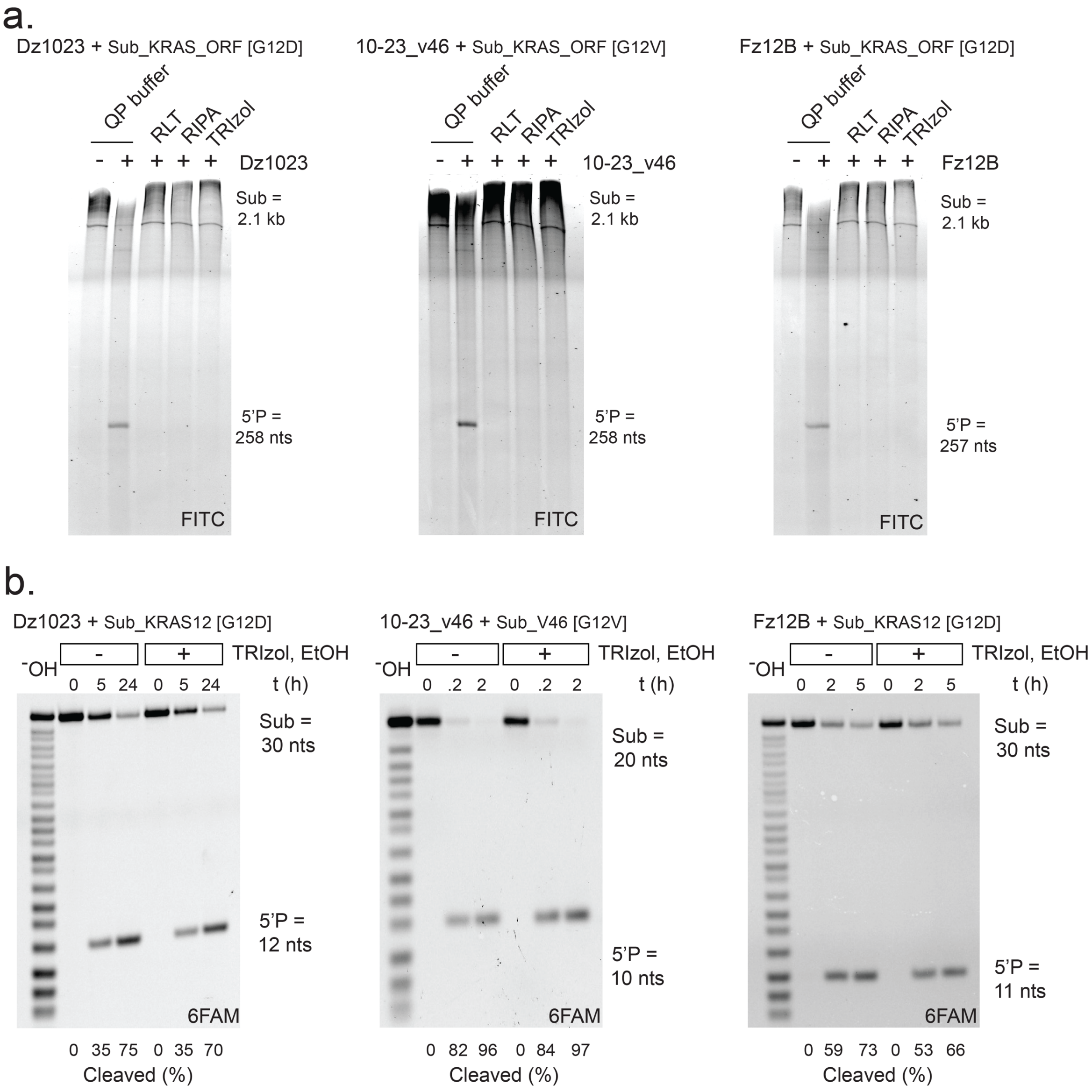

Supplementary Figure 2. RNA-cleaving DNAzyme and XNAzyme oligonucleotide catalysts regain their full activity following treatment with common cell lysis reagents.

*Urea-PAGE gels showing (**a**) KRAS transcripts (1 μM) incubated with oligonucleotide catalysts (5 μM) for 48 h at 25 ^{o}C in common cell lysis reagents as indicated (or quasi-physiological buffer, QP), or (**b**) activity of oligonucleotide catalysts following incubation in Trizol and recovery by ethanol precipitation, measured in QP buffer at 37 ^{o}C with 2.5 μM catalysts and 0.5 μM short RNA substrates. ($^-$OH) = partially hydrolysed RNA substrate.*

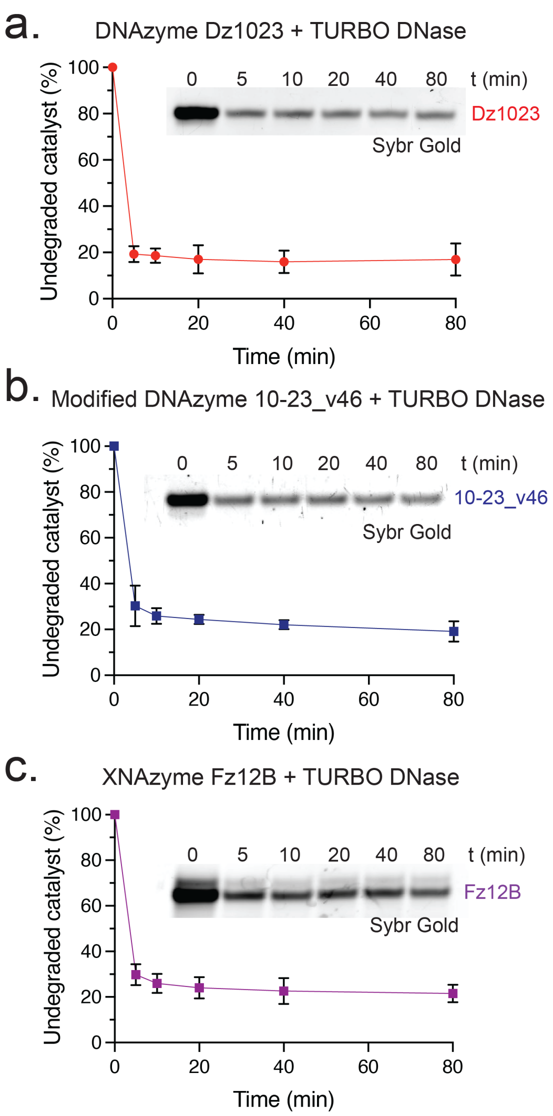

Supplementary Figure 3. Typical reactions using an engineered DNase I with enhanced activity plateau without fully degrading DNAzyme and XNAzyme catalysts.

*Graphs and example Urea-PAGE gels stained with Sybr Gold stain to visualise oligonucleotide catalysts (1 μM) treated with TURBO DNase (Invitrogen / Thermo Fisher Scientific) at 37 ^{o}C for the times indicated; (**a**) unmodified DNAzyme Dz1023, (**b**) modified DNAzyme 10-23_v46, and (**c**) XNAzyme Fz12B. Data and error bars represent mean $\pm$SEM of three independent experiments.*

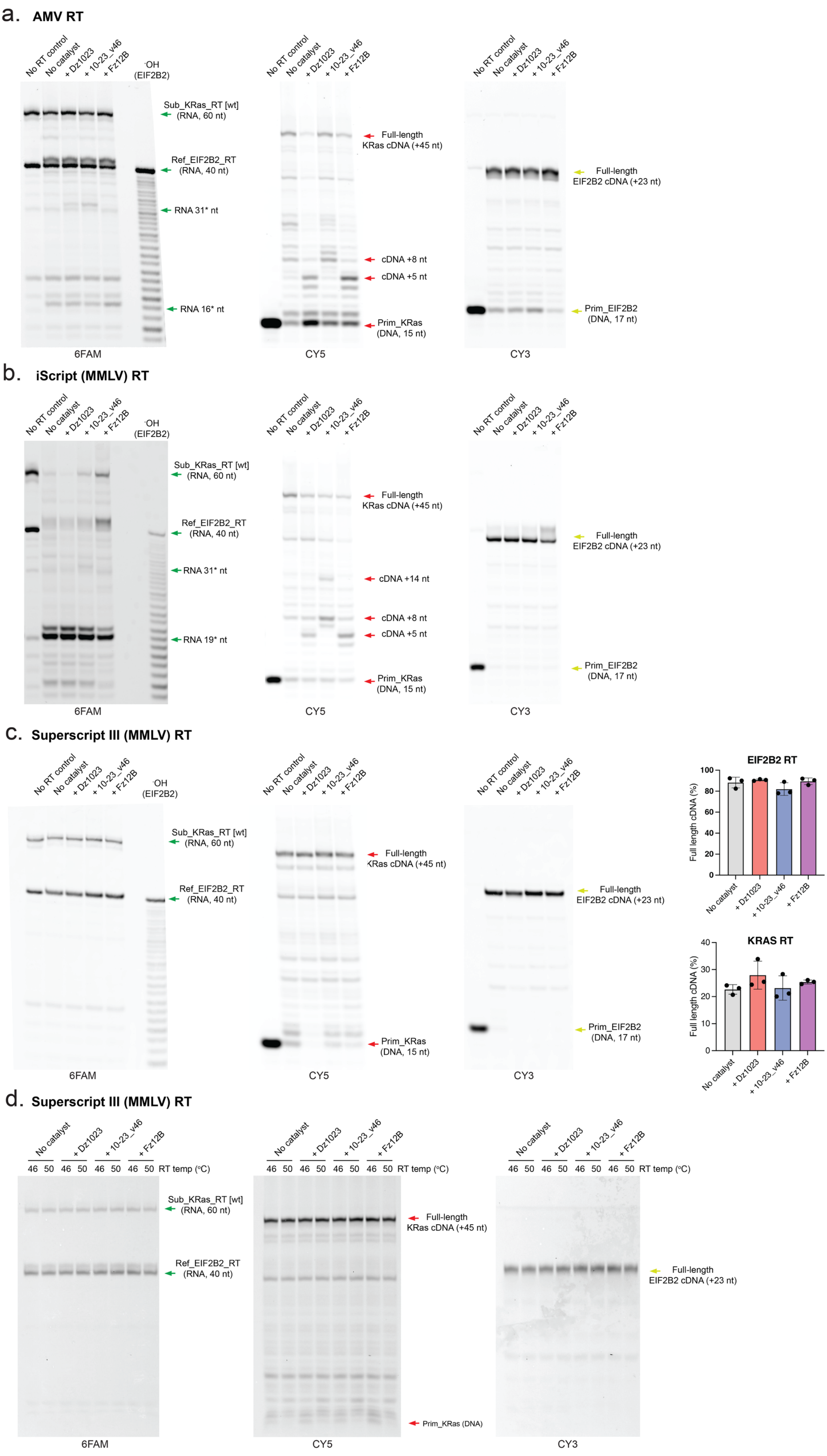
a. AMV RT
No RT control
No catalyst
+ Dz1023
+ 10-23_v46
+ Fz12B
-OH (EIF2B2)
Sub_KRas_RT [wt] (RNA, 60 nt)
Ref_EIF2B2_RT (RNA, 40 nt)
RNA 31* nt
RNA 16* nt
6FAM
Full-length KRas cDNA (+45 nt)
cDNA +8 nt
cDNA +5 nt
Prim_KRas (DNA, 15 nt)
CY5
Full-length EIF2B2 cDNA (+23 nt)
Prim_EIF2B2 (DNA, 17 nt)
CY3
b. iScript (MMLV) RT
RNA 19* nt
cDNA +14 nt
c. Superscript III (MMLV) RT
EIF2B2 RT
Full length cDNA (%)
KRAS RT
d. Superscript III (MMLV) RT
46 50 46 50 46 50 46 50 RT temp (°C)
Prim_KRas (DNA)

Supplementary Figure 4. RT reactions with RNase H-active RT polymerases show template-specific stalling of cDNA synthesis when spiked with DNAzyme or XNAzyme catalysts.

*Urea-PAGE gels showing short RNA reverse transcription assays comprising 0.1 μM 6FAM-labelled RNA templates Sub_KRAS_RT [wt] (60 nt) or Ref_EIF2B2_RT (40 nt), and template-specific primers 5'-Cy5-labelled Prim_KRAS and 5'-Cy3-labelled Prim_EIF2B2 (0.1 μM each), and either (**a**) AMV, (**b**) iScript (MMLV; RNase H+) or (**c,d**) Superscript III (MMLV; RNase H-) RT polymerase, using (**a,b,c**) their respective manufacturers' recommended conditions (see Materials and Methods), or (**d**) 'Superscript' conditions with reduced reaction temperatures. Reactions were spiked with either 0.1 μM unmodified DNAzyme Dz1023, modified DNAzyme 10-23_v46, XNAzyme Fz12B or buffer alone. Note that although the Sub_KRAS_RT RNA comprises the catalysts' target site, it is the wild-type sequence (KRAS c.35G), so catalysts will bind but have little to no intrinsic cleavage activity in order to exclude this from the assay. Bar charts show full-length cDNA quantified from PAGE gels, as a percentage of extended primer; bars and errors represent mean full-length cDNA $\pm$ SEM in three independent experiments. ($^{-}$OH) indicates partially alkaline hydrolysed RNA used as a molecular weight marker; (*) indicates fragments with apparent mobility +1 nt due to the presence of 3' cyclic phosphates.*

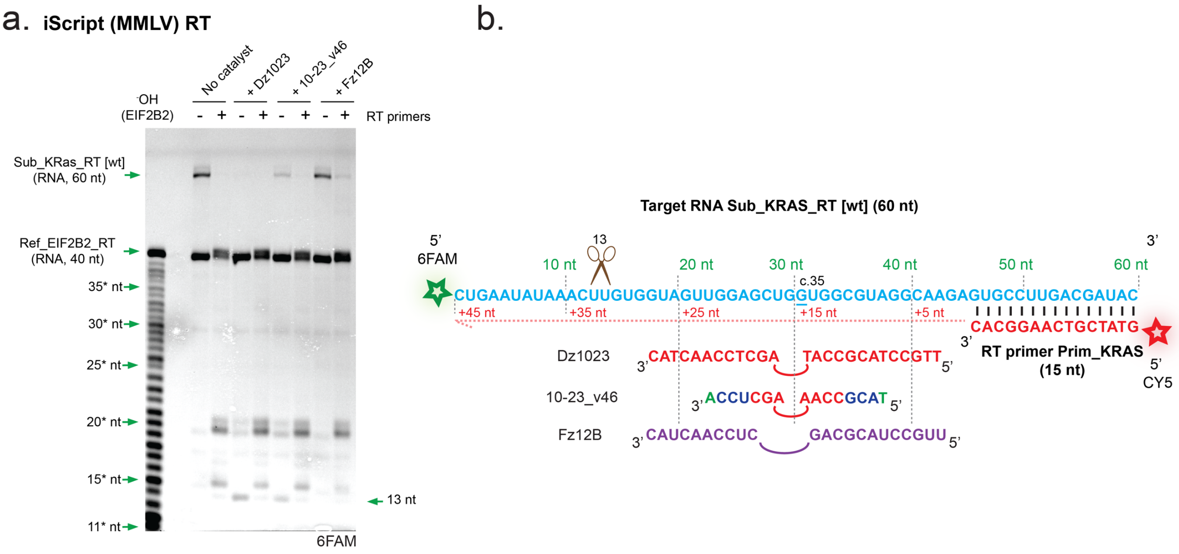

Supplementary Figure 5. DNAzyme catalysts trigger RT polymerase RNase H activity.

*(**a**) Urea-PAGE gel showing short RNA reverse transcription assays comprising 0.1 µM 6FAM-labelled RNA templates Sub_KRAS_RT [wt] (60 nt) or Ref_EIF2B2_RT (40 nt), with or without template-specific primers 5'-Cy5-labelled Prim_KRAS and 5'-Cy3-labelled Prim_EIF2B2 (0.1 µM each), and spiked with either 0.1 µM unmodified DNAzyme Dz1023, modified DNAzyme 10-23_v46, XNAzyme Fz12B or buffer alone, performed using iScript (MMLV; RNase H+) RT polymerase using the manufacturers' recommended conditions (see Materials and Methods). (**b**) Diagram showing complementarity between Sub_KRAS_RT RNA and the oligo catalysts. Scissors indicate deduced RTpol cleavage site in reactions lacking primers but spiked with DNAzymes. (-OH) indicates partially alkaline hydrolysed RNA used as a molecular weight marker; (*) indicates fragments with apparent mobility +1 nt due to the presence of 3' cyclic phosphates.*

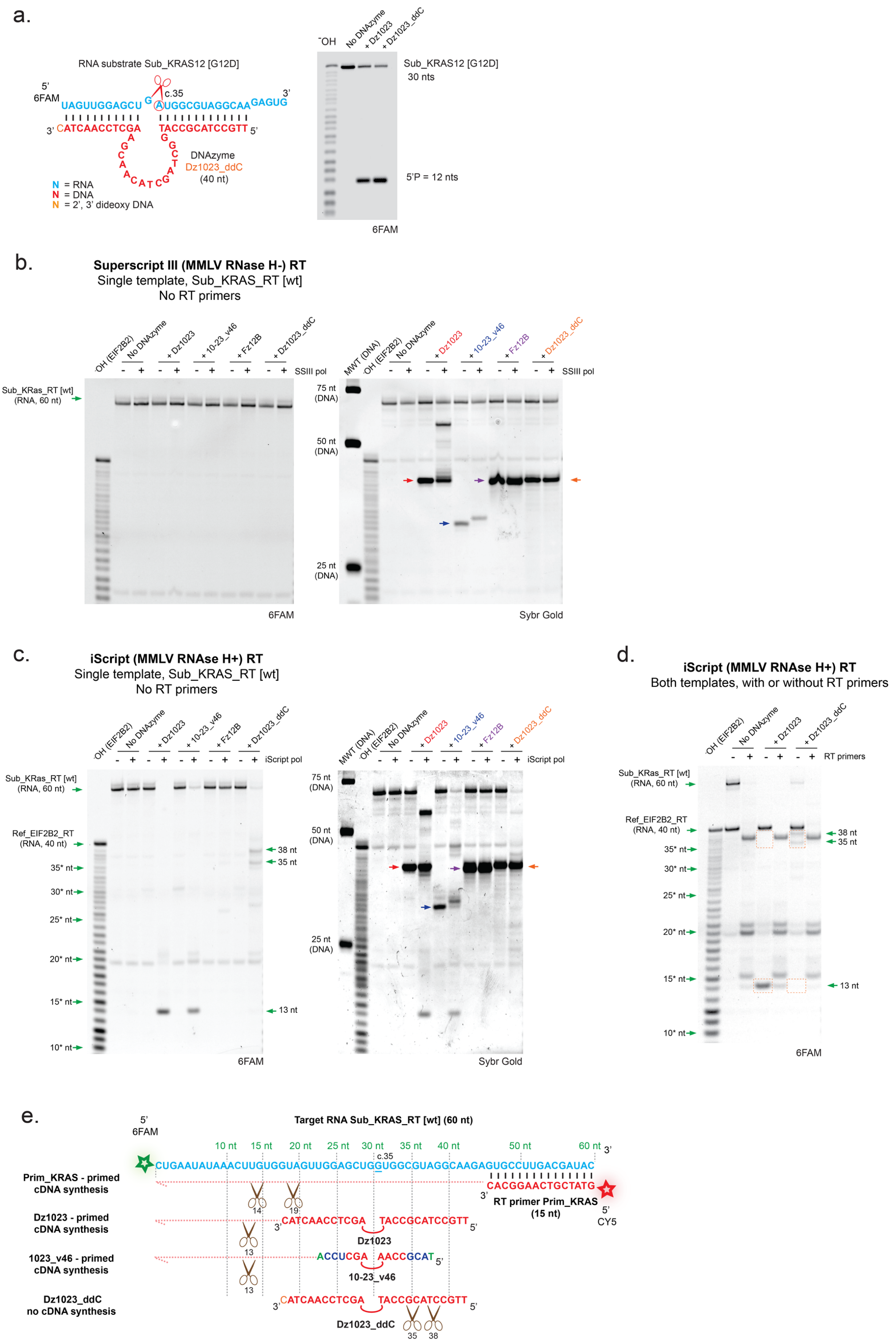

a.
RNA substrate Sub_KRAS12 [G12D]
5'
6FAM
UAGUUGGAGCU G AUGGCGUAGGCAA GAGUG
3'
c.35
3' CATCAACCTCGA TACCGCATCCGTT 5'
DNAzyme
Dz1023_ddC
(40 nt)
N = RNA
N = DNA
N = 2', 3' dideoxy DNA
⁻OH
No DNAzyme
+ Dz1023
+ Dz1023_ddC
Sub_KRAS12 [G12D]
30 nts
5'P = 12 nts
6FAM
b.
Superscript III (MMLV RNase H-) RT
Single template, Sub_KRAS_RT [wt]
No RT primers
OH (EIF2B2)
No DNAzyme
+ Dz1023
+ 10-23_v46
+ Fz12B
+ Dz1023_ddC
SSIII pol
Sub_KRas_RT [wt]
(RNA, 60 nt)
MWT (DNA)
75 nt
(DNA)
50 nt
(DNA)
25 nt
(DNA)
6FAM
Sybr Gold
c.
iScript (MMLV RNAse H+) RT
Single template, Sub_KRAS_RT [wt]
No RT primers
iScript pol
Sub_KRas_RT [wt]
(RNA, 60 nt)
Ref_EIF2B2_RT
(RNA, 40 nt)
35* nt
30* nt
25* nt
20* nt
15* nt
10* nt
38 nt
35 nt
13 nt
6FAM
Sybr Gold
d.
iScript (MMLV RNAse H+) RT
Both templates, with or without RT primers
RT primers
6FAM
e.
Target RNA Sub_KRAS_RT [wt] (60 nt)
5'
6FAM
10 nt
15 nt
20 nt
25 nt
30 nt
35 nt
40 nt
50 nt
60 nt
c.35
CUGAAUAUAAACUUGUGGUAGUUGGAGCUGGUGGCGUAGGCAAGAGUGCCUUGACGAUAC
3'
CACGGAACTGCTATG
Prim_KRAS - primed cDNA synthesis
RT primer Prim_KRAS (15 nt)
5'
CY5
14
19
Dz1023 - primed cDNA synthesis
CATCAACCTCGA TACCGCATCCGTT
Dz1023
13
1023_v46 - primed cDNA synthesis
ACCUCGA AACCGCAT
10-23_v46
Dz1023_ddC no cDNA synthesis
CATCAACCTCGA TACCGCATCCGTT
Dz1023_ddC
35
38

Supplementary Figure 6. DNAzyme catalysts act as primers during RT and generate substrates for RT polymerase RNase H activity.

*(**a**) Sequences and Urea-PAGE gel showing 2',3'-dideoxy-modified DNAzyme Dz1023_ddC (5 μM) cleaving 6FAM-labelled short KRAS RNA (1 μM) in a single-turnover reaction in quasi-physiological conditions (15 h). (**b,c**) Urea-PAGE gels (imaged for 6FAM fluorescence or subsequently stained and imaged for all nucleic acids using Sybr Gold stain) showing short RNA reverse transcription assays comprising 0.1 μM 6FAM-labelled RNA template Sub_KRAS_RT [wt] (60 nt) without template-specific primers, and spiked with either 0.1 μM unmodified DNAzyme Dz1023, modified DNAzyme 10-23_v46, XNAzyme Fz12B or DNAzyme Dz1023_ddC, with or without (**c**) Superscript (MMLV; RNase H-) or (**c**) iScript (MMLV; RNase H+) RT polymerases. (**d**) Urea-PAGE gel (imaged for 6FAM fluorescence) showing short RNA reverse transcription assays performed by iScript RT polymerase comprising 0.1 μM 6FAM-labelled RNA templates Sub_KRAS_RT [wt] (60 nt) and Ref_EIF2B2_RT (40 nt), with or without 0.1 μM template-specific primers Prim_KRAS and Prim_EIF2B2 (0.1 μM each), and spiked with either 0.1 μM unmodified DNAzyme Dz1023, modified DNAzyme 10-23_v46, XNAzyme Fz12B or DNAzyme Dz1023_ddC. (**e**) Diagram showing complementarity between Sub_KRAS_RT RNA and the oligo catalysts and sites of cleavage by RTpol RNase H activity (represented by scissors) generated by DNAzyme-primed cDNA synthesis. 3' blocked Dz1023_ddC cannot prime cDNA synthesis and reveals RNase H activity can also be recruited by the DNAzyme binding arms. ($^{-}$OH) indicates partially alkaline hydrolysed RNA used as a molecular weight marker; (*) indicates fragments with apparent mobility +1 nt due to the presence of 3' cyclic phosphates.*

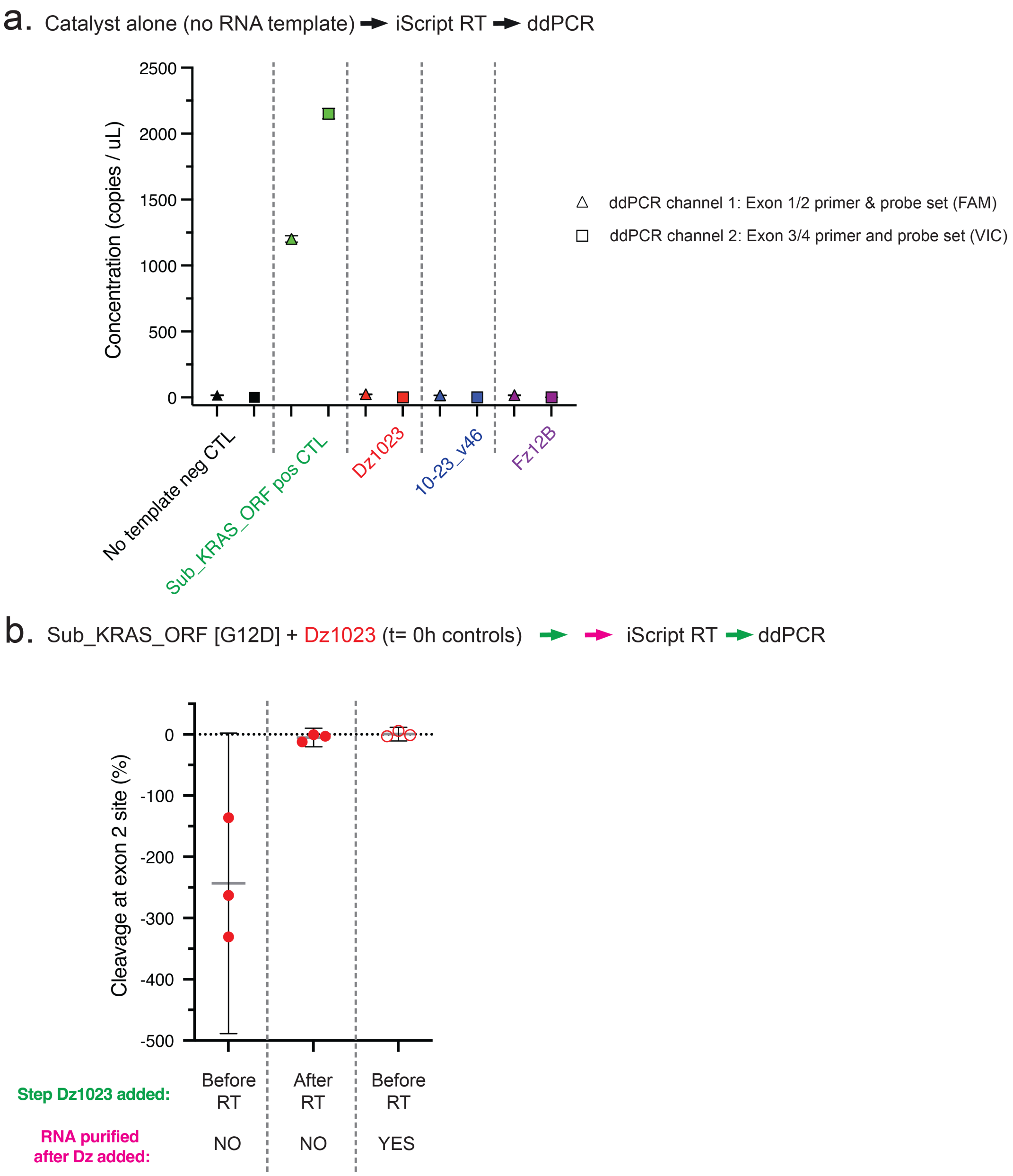

Supplementary Figure 7. Control reactions for KRAS RT-ddPCR assay confirm that the DNAzyme-dependent mismeasurement effect derives from the RT step and not the PCR.

*Grouped scatter graphs showing droplet digital quantitative PCR (ddPCR) measurements using Taqman assays for KRAS exon 1/2 (Hs00364282_m1 (FAM)) and exon 3/4 (Hs00364284_g1 (VIC)) following reverse transcription reactions (5 min at 25 °C, 20 min at 46 °C) performed with iScript RT polymerase; (**a**) no-RNA-template RTs, spiked with (5 μM) unmodified DNAzyme Dz1023, modified DNAzyme 10-23_v46 or XNAzyme Fz12B, showing that the catalysts are not themselves detected by the ddPCR assay; (**b**) RTs with (1 μM) Sub_KRAS_ORF [G12D] RNA spiked with (5 μM) unmodified DNAzyme Dz1023 before or after the RT reaction, with or without subsequent RNA purification prior to RT (see Materials and Methods), showing that the false negative cleavage effect does not occur if Dz1023 is added after RT or if RNA spiked with Dz1023 is repurified prior to RT.*

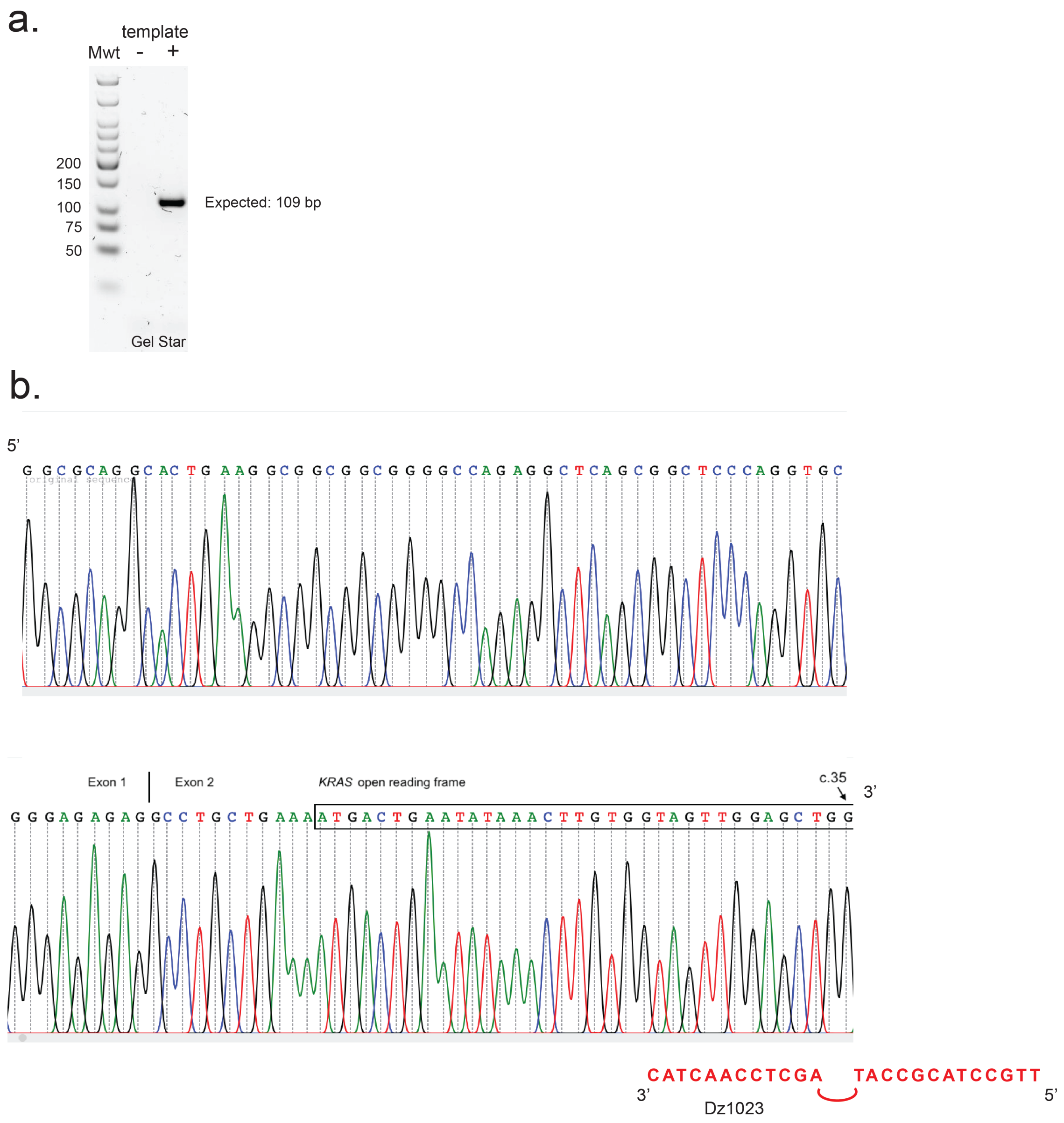

Supplementary Figure 8. The KRAS exon 2 TaqMan qPCR assay amplicon.

***(a)** 4% agarose gel showing non-quantitative PCR primed used KRAS exon 1/2 Taqman assay Hs00364282_m1 and templated using KRAS ORF plasmid (Origene, SC109374). (**b**) Chromatogram showing Sanger sequencing of TOPO-TA-cloned PCR amplicon produced by Taqman KRAS exon 1/2 assay Hs00364282_m1. Sequencing reveals that the reverse primer in this assay must overlap the sequence of the 3' binding arm of the Dz1023 DNAzyme, suggesting that DNAzymes acting as primers during RT-PCR will generate cDNA product that will be detected by this assay.*

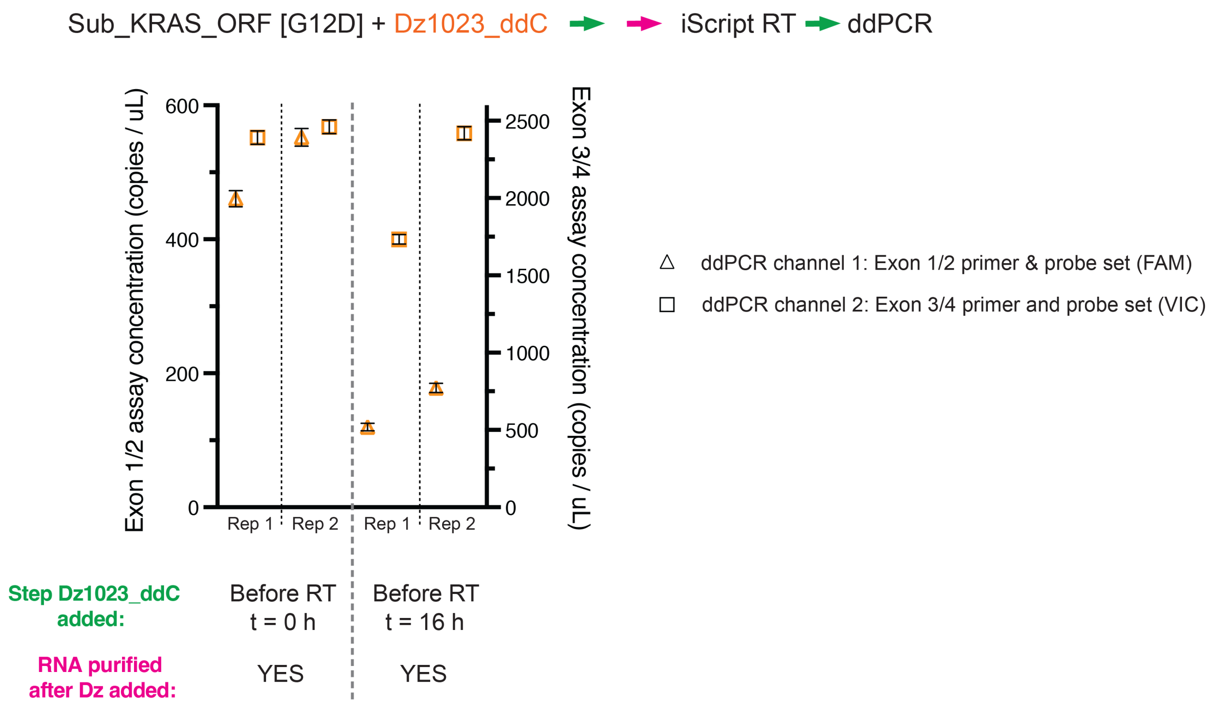

Supplementary Figure 9. Control reactions for KRAS RT-ddPCR assay confirm that the DNAzyme-dependent mismeasurement effect derives from the RT step and not the PCR.

Grouped scatter graph showing droplet digital quantitative PCR (ddPCR) measurements using Taqman assays for KRAS exon 1/2 (Hs00364282_m1 (FAM)) and exon 3/4 (Hs00364284_g1 (VIC)) following reverse transcription reactions (5 min at 25 °C, 20 min at 46 °C) performed with iScript RT polymerase. (1 μM) Sub_KRAS_ORF [G12D] RNA spiked with (5 μM) DNAzyme Dz1023_ddC, allowed to cleave (t = 16 h) or not (t = 0 h) in QP buffer, then purified prior to RT. This shows an example of detection of bona fide cleavage - the Taqman exon 1/2 assay signal is reduced (here by ~65%) relative to the exon 3/4 assay signal following incubation of substrate with catalyst, which is removed by purification of RNA prior to RT-ddPCR.

Name	Sequence (5′ → 3′)	Modifications	Notes
10-23_KRasC[13+12] aka Dz1023	TTGCCTACGCCATGGCTAGCTACAACGAA GCTCCAACTAC	none	Variant of the 10-23 DNAzyme[1, 2] designed to pair with *KRAS* mRNA residues c.22-c.47 and cleaves between c.34 and c.35 when c.35 is an A (KRAS G12D)
Dz1023_ddC	TTGCCTACGCCATGGCTAGCTACAACGAA GCTCCAACTA**[ddC]**	2',3'-dideoxy-C chain terminator	Variant of Dz1023 lacking a terminal 3'O to prevent 3' extension
10-23_v46	**[LNA-T][mA][mC][mG]**CCAA*GGCTAG**[mC][mU]**ACAAC***[MOE-G]**AAGC**[mU][mC][mC][LNA-A]**	LNA (green), 2'OMe-RNA (dark blue), 2'MOE-RNA (brown), phosphorothioate (*)	A modified variant of the 10-23 DNAzyme[3] designed to pair with *KRAS* mRNA residues c.27-c.41 and cleaves between c.34 and c.35 when c.35 is a U (KRAS G12V)
Fz12B aka FR6_1_KRas12B	**fU*fU*fG*fCfCfUfAfCfGfCfAfGfGfCfAfCfGfG fUfUfUfGfAfUfUfUfCfCfGfUfCfUfCfCfAfAfC* fU*fA*fC**	FANA (purple), phosphorothioate (*)	A variant of the FR6_1 FANAzyme[2] designed to pair with *KRAS* mRNA residues c.22-c.47 and cleave between c.33 and c.34 when c.35 is an A (KRAS G12D)
Sub_KRas12 [G12D]	[6FAM]rUrArGrUrUrGrGrArGrCrUrGrArUrGrGr CrGrUrArGrGrC	5'-6FAM, RNA (cyan)	30 nt RNA equivalent to *KRAS* [G12D] residues c.23-c.45; substrate for Dz1023 and Fz12B described in ref [2]
Sub_V46 [G12V]	[6FAM]rGrUrUrGrGrArGrCrUrGrUrUrGrGrCrG rUrArGrG	5'-6FAM, RNA (cyan)	20 nt RNA equivalent to *KRAS* [G12V] residues c.25-c.44; substrate for 10-23_v46 described in ref [3]
LC	[CY5]AAAAAAACCCCCAAAACCCCCCAAAA CCCCAAAACCCCAAAACCCCAAAA	5'-Cy5	Loading control oligo used in Urea-PAGE assays of transcript cleavage
Sub_KRAS_RT [wt]	[6FAM]rCrUrGrArArUrArUrArArArCrUrUrGrUr GrGrUrArGrUrUrGrGrArGrCrUrGrGrUrGrGrCr GrUrArGrGrCrArArGrArGrUrGrCrCrUrUrGrAr CrGrArUrArC	5'-6FAM, RNA (cyan)	60 nt RNA equivlant to *KRAS* [wt] residues c.5-c.64; bound but non-(or poorly-)cleavable substrate for catalysts, used as template in 'short RNA' RT assay
Ref_EIF2B2_RT	[6FAM]rCrArArCrUrCrCrArGrUrCrCrArArCrAr UrCrArUrUrGrArGrGrCrGrArUrUrArArUrGrArG rCrUrGrC	5'-6FAM, RNA (cyan)	40 nt RNA equivalent to *EIF2B2* residues c.382-c.421; not bound by catalysts, used as template in 'short RNA' RT assay
Prim_KRAS	[CY5]GTATCGTCAAGGCAC	5'-Cy5	RT primer complementary to Sub_KRAS_RT [wt] RNA template
Prim_EIF2B2	[CY3]GCAGCTCATTAATCGCC	5'-Cy3	RT primer complementary to Ref_EIF2B2_RT RNA template

Supplementary Table 1. Oligonucleotide sequences used in the study.

Materials and methods

All chemicals were supplied by Merck / MilliporeSigma (Germany) unless stated otherwise.

Oligonucleotide synthesis. All oligonucleotides were synthesised by Integrated DNA Technologies (Belgium) or Merck / MilliporeSigma (Germany) except "Fz12B", which was prepared in house using a Mermade 4 (BioAutomation / LGC Biosearch, USA) under Argon N5.0 (BOC UK), using 1 µmole scale Universal Support (DMT off, N-iPr) 1000Å CPG columns (LGC Biosearch, USA) and phosphoramidites of 2'-F-arabinonucleosides (FANA) (Glen Research, USA). Phosphoramidites were prepared as 0.1M solutions in anhydrous acetonitrile (ACN)(LGC Biosearch, USA) and activated with 0.25M 5-ethylthio-1H-tetrazole (ETT)(LGC Biosearch, USA) in ACN. Deblocking was performed with 3% dichloroacetic acid in toluene (LGC Biosearch, USA), capping of failure sequences was performed with a 1:1 mix of Cap B (16% 1-methylimidazole in THF)(LGC Biosearch, USA) and CapA (THF/lutidine/acetic anhydride (8:1:1)(LGC Biosearch, USA), and oxidation was performed with 0.02M iodine THF/pyridine/water (89.6:0.4:10)(LGC Biosearch, USA). Two consecutive 900 s couplings were performed for all phosphoramidites. Deprotection and cleavage from the CPG support was achieved by incubation in 3:1 NH_4OH:EtOH for 48 h at room temperature, which was subsequently evaporated by speedvac. Oligos were resuspended in water, diluted in formamide PAGE loading buffer and purified by Urea-PAGE.

Analysis of oligonucleotides mass by MALDI-TOF mass spectrometry. MALDI-TOF mass spectrometry was performed using a three-layer sandwich method. 0.5 µL of matrix solution (48 mg/mL 2',4',6'-trihydroxyacetophenone, 24 mg/mL 2',3',4'-trihydroxyacetophenone (Alfa Aesar / Thermo Scientific, USA) and 10 mg/mL di-ammonium hydrogen citrate in 50:50 ACN:water) was spotted onto the MALDI target plate, followed by 0.5 µL of oligonucleotide sample (5–25 µM in water), and an additional 0.5 µL of matrix solution. The spots were left to dry between each addition. Mass spectrometric measurements were performed in linear negative-ion mode using a Shimadzu Biotech Axima Performance MALDI TOF/TOF mass spectrometer (Shimadzu, Kyoto, Japan).

Synthesis and labelling of long KRAS transcript RNA. "Sub_KRAS_ORF" RNA (2.1 kb) was prepared as described previously[2]. Briefly, human *KRAS* (NM_004985) ORF dsDNA templates encoding mutants c.35G>A [G12D] or c.35G>T [G12V] were generated from the ORF clone (Origene, SC109374) in plasmid pCMV6-XL6 by site-directed mutagenesis using a QuikChange II kit (Agilent Technologies, USA)). Plasmid DNA was linearised with Cfr9I / XmaI (Thermo Fisher Scientific, USA) and used to prepare RNA by in vitro transcription using a HiScribe SP6 kit (NEB, USA). Following transcription, reactions were treated with TURBO DNase (Thermo Fisher Scientific, USA) and RNA purified using an RNA Clean & Concentrator-5 kit (Zymo Research, USA). 5' labelling was performed using Fluorescein-5-maleimide (Invitrogen) and the 5' EndTag DNA/RNA Labelling Kit (VectorLabs), including an additional 3 h incubation at room temperature. Labelled RNA was re-purified by TRIzol/chloroform extraction and an RNA Clean & Concentrator-5 kit.

Denaturing poly-acrylamide gel electrophoresis. Urea-PAGE gels were prepared with 8 M urea in TBE buffer, 15 % (w/v) acrylamide:bis-acrylamide 19:1 (Severn Biotech, UK), 0.05 % w/v ammonium persulphate (APS), and 0.2 % v/v N,N,N',N' tetramethylethylenediamine (TEMED). Gels were run in TBE buffer at 10 W for 10 minutes followed by 24 W for 1.5-2 hours. PAGE loading buffer was 95 % (v/v) formamide, 10 mM Tris-HCl pH 7.4, 50 mM EDTA, and 0.01 % (w/v) bromophenol blue,

and the samples were heated to 90 °C for 10 minutes prior to loading. DNA (Low Molecular Weight ladder N3233 (New England Biolabs, USA)) or partially hydrolysed 5'-6FAM labelled RNAs were used as markers. Gels were imaged using an FLA-5000 fluorescence gel imager (Fujifilm, Japan) using FITC/FAM, Cy3 or Cy5 settings, and, where indicated, subsequently stained with SYBR Gold nucleic acid stain (Thermo Fisher Scientific, USA). For recovery of oligos from PAGE gels, excised slices were pulverised using 5 mm nuclease-free stainless-steel ball bearings (Qiagen, Germany) in a TissueLyser bead mill (Qiagen, Germany). Nuclease-free water (Qiagen, Germany) was added to create a slurry, which was freeze-thawed using dry ice and a 99 °C heat block, then incubated on a rotary mixer for ~16 h at room temperature and supernatant recovered using 0.45 µm spin filters (Spin-X, Corning cat. no. 8163). Oligos were precipitated in ice cold ethanol and pellets washed with 70% ethanol and dried by speedvac.

RNA cleavage reactions. All cleavage reactions were performed using a 5-fold excess of catalytic oligo (5 µM) relative to substrate RNA (1 µM) unless stated otherwise, with a prior annealing step (80 °C, 1 min) in nuclease-free water (Qiagen, Germany) and reactions terminated by addition of excess loading buffer followed by snap-freezing on dry ice and storage at -80 °C. Reactions under quasi-physiological conditions were incubated at 37 °C in QP buffer (30 mM EPPS pH 7.4, 150 mM KCl, 1 mM $MgCl_2$); reactions under lysis conditions were incubated at 25 °C in RLT buffer (Qiagen), TRIzol (Zymo Research), or RIPA lysis buffer (Sigma / Merck); reactions under DNase conditions were incubated at 37 °C in DNase I buffer (10 mM Tris-HCl, pH 7.6, 2.5 mM $MgCl_2$, 0.05 mM $CaCl_2$); reactions under RT conditions were incubated at 46 °C in iScript buffer (Bio-Rad, USA) or 50 °C Superscript III first-strand buffer (1X: 50 mM Tris-HCl, pH 8.3, 75 mM KCl, 3 mM $MgCl_2$)(ThermoFisher Scientific, USA).

DNAzyme and XNAzyme single-turnover reaction rates. Pseudo-first-order reaction rates for catalysts were determined under single-turnover pre-steady-state conditions ($[E] > [S] \gg K_m$), from three independent RNA cleavage reactions using either 5'-6FAM-labelled 20 or 30 nt RNA substrates or 2.1 kb 5'-FITC-labelled Sub_KRAS_ORF RNA, assayed by densitometry of Urea-PAGE gels scanned using a fluorescence imager (FLA-5000, Fujifilm, Japan). For 20 nt or 30 nt RNA reactions, cleavage was determined by measurement of both 5' product and uncleaved substrate at each timepoint. For 2.1 kb transcript RNAs (as the uncleaved RNAs resolve poorly by PAGE), the appearance of 5' cleavage products over time was determined, relative to the reaction endpoint (arbitrarily defined as 100%) and normalised to loading controls (an irrelevant Cy5-labelled DNA oligo, LC, was spiked into each reaction). The observed rate constant (k_{obs}) was calculated by non-linear regression to a one-phase association (with the origin constrained to 0) in GraphPad Prism v10.

DNase digestion assay. Catalysts (1 µM) were incubated with 0.1 U/µl DNase I (NEB, USA) in DNase I buffer or 0.1 U/µl TURBO DNase in 1× TURBO buffer (Thermo Fisher Scientific, USA) at 37 °C for up to 80 minutes. Reaction samples were terminated by the addition of loading buffer followed by snap-freezing on dry ice and storage at -80°C prior to analysis by Urea-PAGE

Short RNA reverse transcription (RT) assays. RT reactions were performed using 5'-6FAM-labelled RNAs 60-nt Sub_KRAS_RT [wt] (0.1 µM) or 40-nt Ref_EIF2B2_RT (0.1 µM), with or without catalysts (0.1 µM) and/or RT primers (5'-Cy5-labelled Prim_KRAS and 5'-Cy3-labelled Prim_EIF2B2, 0.1 µM each), using either iScript (Bio-Rad), AMV (Promega, USA) or SuperScript III (Thermo Fisher Scientific, USA) reverse transcriptases, using the appropriate manufacturer-

supplied buffer and protocol; iScript (Bio-Rad) reactions used 0.05 µl polymerase per µl reaction in 1× iScript Select Reaction Mix and 0.1 µl GSP enhancer (Bio-Rad) per µl reaction and incubated at 25 °C for 5 min, 46 °C for 20 min, followed by 85 °C for 5 min; AMV (Promega, USA) reactions used 1.2 U/µl polymerase in 1× AMV Reverse Transcriptase Reaction Buffer (50 mM Tris-HCl pH 8.3, 50 mM KCl, 10 mM $MgCl_2$, 10 mM DTT), 0.5 mM dNTPs, and incubated at 42 °C for 60 min, followed by 95 °C for 5 min; SuperScript III reactions used 10 U/µL polymerase in 1× Superscript First-Strand buffer with 5 mM DTT, 0.5 mM dNTPs, 2 U/µL reaction RNaseOUT, and incubated at 50 °C for 60 min, followed by 70 °C for 15 min.

Transcript RNA reverse transcription droplet digital PCR (RT-ddPCR) assays. RNAs Sub_KRAS_ORF [G12D] (1 µM) or Sub_KRAS_ORF [G12V] (1 µM) were spiked with catalysts (1 µM unless stated otherwise)(G12D: Dz1023, Dz1023_ddC or XNAzyme Fz12B, G12V: 10-23_v46), but immediately used for RT (i.e. without a cleavage reaction incubation). RT reactions were performed using an iScript cDNA synthesis kit (Bio-Rad, USA) according to the manufacturer's instructions, using 400 ng Sub_KRAS_ORF RNA per 10 µl reaction. cDNA was diluted 1:200,000 and used as template for droplet digital PCR (ddPCR), using 2 µl per 20 µl reaction. Emulsion PCRs were prepared, cycled (95°C for 10 min, 40 x [94°C for 30 s, 58°C for 1 min (1°C /s ramp rate)], 98°C for 10 min) and analysed using a QX200 system (Bio-Rad, USA). Data were processed using QuantaSoft software (Bio-Rad). Apparent cleavage at or close to the catalysts' binding site was determined using multiplexed TaqMan assays (Thermo Fisher Scientific, USA) as described previously[2]. Assay Hs00364282_m1 (FAM) spans the exon 1–2 junction and includes the catalyst target site, and assay Hs00364284_g1 (VIC), spans the exon 3–4 junction, which is not targeted by the catalysts; the quantity of FAM-positive droplets were normalised to the VIC-positive droplets in the same sample, and double-referenced to matched control reactions without catalysts.

RNA purification to remove catalytic oligos. RNA purification was performed to mimic a typical cellular RNA workup. Catalyst-spiked RNA samples were diluted with TRIzol reagent (Zymo Research, USA) and chloroform to obtain an aqueous phase that was further purified using an RNA Clean & Concentrator-5 kit (Zymo Research, USA) following the manufacturer's 'small RNA removal' protocol followed by an on-column DNase I digestion step (room temp, 15 min) and an additional wash, which we have previously found was necessary to deplete catalysts below PAGE-detectable levels using RNA purification columns[2].

Cloning and Sanger sequencing. The amplicon generated in a PCR using Onetaq (NEB, USA) templated by the *KRAS* ORF plasmid (Origene, SC109374) and primed using the KRAS Exon 1/2 Taqman assay (Hs00364282_m1) was purified by agarose gel electrophoresis, recovered using a gel extraction kit (Qiagen, Germany) and cloned into pCR4-TOPO using a TOPO TA Cloning Kit (Invitrogen / Thermo Fisher Scientific, USA), according to the manufacturer's instructions. Successful transformants were cultured in 2XTY with ampicillin, plasmids extracted using a Monarch Plasmid Miniprep Kit (New England Biolabs, USA) and Sanger sequenced by Source BioScience (UK) using M13R primers.

Supplementary References

1. S. W. Santoro and G. F. Joyce, *Proceedings of the National Academy of Sciences of the United States of America*, 1997, **94**, 4262-4266.
2. A. I. Taylor, J. K. Wan, M. J. Donde, S.-Y. Peak-Chew and P. Holliger, *Nature Chemistry*, 2022, **14**, 1295-1305.
3. K. Nguyen, T. N. Malik and J. C. Chaput, *Nat Commun*, 2023, **14**, 2413.
